# Androgen-mediated maternal effects and trade-offs: postnatal hormone development, growth, and survivorship in wild meerkats

**DOI:** 10.3389/fendo.2024.1418056

**Published:** 2024-09-30

**Authors:** Charli S. Davies, Caroline L. Shearer, Lydia K. Greene, Jessica Mitchell, Debbie Walsh, Vivian C. Goerlich, Tim H. Clutton-Brock, Christine M. Drea

**Affiliations:** ^1^ Department of Evolutionary Anthropology, Duke University, Durham, NC, United States; ^2^ Kalahari Research Trust, Kuruman River Reserve, Northern Cape, South Africa; ^3^ University Program in Ecology, Duke University, Durham, NC, United States; ^4^ Department of Zoology, University of Cambridge, Cambridge, United Kingdom; ^5^ Mammal Research Institute, University of Pretoria, Pretoria, South Africa; ^6^ Department of Biology, Duke University, Durham, NC, United States

**Keywords:** female masculinization, flutamide, IGF-1, life-history trade-offs, ontogeny, prenatal programming, sex steroids, sexual differentiation

## Abstract

**Introduction:**

Mammalian reproductive and somatic development is regulated by steroid hormones, growth hormone (GH), and insulin-like growth factor-1 (IGF-1). Based largely on information from humans, model organisms, and domesticated animals, testosterone (T) and the GH/IGF-1 system activate sexually differentiated development, promoting male-biased growth, often at a cost to health and survivorship. To test if augmented prenatal androgen exposure in females produces similar developmental patterns and trade-offs, we examine maternal effects in wild meerkats (*Suricata suricatta*), a non-model species in which adult females naturally, albeit differentially by status, express exceptionally high androgen concentrations, particularly during pregnancy. In this cooperative breeder, the early growth of daughters predicts future breeding status and reproductive success.

**Methods:**

We examine effects of normative and experimentally induced variation in maternal androgens on the ontogenetic patterns in offspring reproductive hormones (androstenedione, A_4_; T; estradiol, E_2_), IGF-1, growth from pup emergence at 1 month to puberty at 1 year, and survivorship. Specifically, we compare the male and female offspring of dominant control (DC or high-T), subordinate control (SC or lower-T), and dominant treated (DT or blocked-T) dams, the latter having experienced antiandrogen treatment in late gestation.

**Results:**

Meerkat offspring showed sex differences in absolute T and IGF-1 concentrations, developmental rates of A_4_ and E_2_ expression, and survivorship — effects that were sometimes socially or environmentally modulated. Atypical for mammals were the early male bias in T that disappeared by puberty, the absence of sex differences in A_4_ and E_2_, and the female bias in IGF-1. Food availability was linked to steroid concentrations in females and to IGF-1, potentially growth, and survival in both sexes. Maternal treatment significantly affected rates of T, E_2_, and IGF-1 expression, and weight, with marginal effects on survivorship; offspring of DT dams showed peak IGF-1 concentrations and the best survivorship.

**Discussion:**

Maternal effects thus impact offspring development in meerkats, with associated trade-offs: Whereas prenatal androgens modify postnatal reproductive and somatic physiology, benefits associated with enhanced competitiveness in DC lineages may have initial costs of reduced IGF-1, delay in weight gain, and decreased survivorship. These novel data further confirm the different evolutionary and mechanistic pathways to cooperative breeding and call for greater consideration of natural endocrine variation in both sexes.

## Introduction

Sexual differentiation is a lifelong developmental process whereby the production of male and female phenotypes arises from a complex interplay between genetic, hormonal, and environmental factors (1, 2). The earliest environmental influence on an individual’s underlying biological processes derives from the maternal (or prenatal) milieu. Maternal effects on offspring are widespread and diverse in the animal kingdom, evident in the nongenetically determined, life-history markers of morphological, physiological, and behavioral development, as well as health, survival, and fitness (3). Notably, maternal androgens in egg yolks are well known to impact offspring growth, survival, and immunity in oviparous species (4, 5). In mammals, maternal effects are commonly examined in the context of differences in maternal diet and stress (6, 7), arising from differential access to resources (8) or social instability and conflict (9). They also owe to impacts on the fetal endocrine milieu, particularly via differential exposure of fetal females to androgens (7), be they of fraternal or maternal origin. Here, using a nonmodel system – the meerkat (*Suricata suricatta*) – examined during an environmental stressor (i.e., a drought), we test for effects of naturally and experimentally induced variation in maternal androgens on the endocrine development, growth, and survivorship of offspring.

Our study contributes to a slow-growing literature on the effects of androgens in females, which is related both to the process of sexual differentiation and to potential epigenetic effects of early hormone exposure. Typically, in mammals, androgens of fetal testicular origin are required to produce a temporal sequence of masculinizing effects in XY individuals; when fetal XX individuals are exposed to these androgens, they incur variable, reproductively costly, masculinizing effects depending on the timing, duration, and quantity of exposure (10). In certain litter-bearing species (particularly rodents, lagomorphs, and swine), fetal females are exposed to varying concentrations of testicular hormones owing to their *in utero* distance from male littermates; intrauterine position produces a gradient of female morphotypes, ranging from feminized to masculinized (11–13). Beyond the well-known organizational effects of fraternal androgens on female morphology, physiology, and behavior (e.g., aggression), are the effects of androgens on various life-history traits (14). For instance, fraternally masculinized females often experience reduced growth or weight at weaning, with delayed puberty, as well as reduced fecundity (e.g. disrupted ovarian cycles), fertility (e.g. reduced litter size), and survival (15–18). Even in humans, females with male co-twins can be masculinized (19) and experience reduced fertility (20). Nonetheless, effects are inconsistent across species, may differ between captive and wild populations, and have also been shown to produce some benefits to masculinized females (21, 22). More exceptionally, masculinization can arise routinely in all female members of certain species, such as the spotted hyena [*Crocuta crocuta* (23–25)], ring-tailed lemur [*Lemur catta* (26)], and meerkat (27). In these cases, natural selection operates on prenatal androgens of maternal origin, producing transgenerational effects, ultimately with female social and reproductive benefits that far outweigh any costs. Nonetheless, our understanding of postnatal ontogeny and life-history trade-offs in such species remains limited.

It long has been established that mammalian reproductive, somatic, and skeletal development is regulated, in large part, by the actions of steroid hormones, particularly androgens [e.g (28–30)], with increasing appreciation for the role of estrogens [e.g (31–33)] and genetic factors (2, 23, 34). In early life, activation of the mammalian hypothalamic-pituitary-gonadal (HPG) axis varies depending on the sex and developmental stage of the individual. Notably, the prenatal period is critical for establishing the reproductive phenotype of embryos, as well as their future steroid production and sensitivities. Testosterone (T) is a critical organizing hormone, including in fraternally and maternally masculinized female fetuses; however, one of its androgenic precursors, androstenedione (A_4_), is also implicated in maternally masculinized females and may be capable of producing or contributing to male-like traits without interfering with reproductive function [spotted hyenas: (35–37); ring-tailed lemurs: (26, 38); meerkats (27)]. Across young mammals, there is sometimes only a brief period of HPG activity between birth and preweaning, particularly evident in males (39); otherwise, like juvenility, infancy is generally a period of reproductive quiescence, such that sex hormones may be initially at the lower limits of detectability, increasing as the HPG axis is reactivated when adolescent animals approach puberty (i.e., the pubertal surge). Typically, throughout postnatal development, androgen concentrations are greater in males than in females, whereas the reverse is true of estrogens, particularly estradiol [E_2_; reviewed in (39, 40)].

Only more recently has evidence highlighted (a) the key role of insulin-like growth factor-1 (IGF-1) in these anabolic processes, (b) the integration of different neuroendocrine mechanisms, and (c) the significant trade-offs between reproductive development, growth, and longevity [reviewed in (41–43)]. Principally produced in the liver, IGF-1 is a polypeptide hormone that manages the effects of growth hormone (GH), stimulating somatic and skeletal growth; it is also a powerful regulator of reproduction, promoting steroidogenesis and growth of reproductive structures. As regards neuroendocrine integration, sex steroids are known to modulate the effects of IGF-1, and vice versa; however, androgens and estrogens can exert distinct and opposite effects (33). For instance, T typically raises, whereas E_2_ typically lowers, total IGF-1 concentrations (44). Androgen and free IGF-1 concentrations thus generally correlate positively with somatic male-biased growth, suggesting potentially age-dependent sex differences in concentration [e.g (45)]. Over the course of development in both sexes, IGF-1 concentrations are initially raised and begin to plateau or decrease at or around puberty, with slower cell growth or metabolism in adulthood potentially serving as a physiological strategy for extending lifespan (46). Thus, as with T (47, 48), IGF-1 promotes growth and reproduction at a cost to longevity (43), such that both hormones are key when considering life-history trade-offs.

The meerkat – an aggressive (49, 50), social mongoose and obligate cooperative breeder – has emerged as an important system for examining the behavioral and reproductive effects of androgens in females (27, 51, 52). Notably, dominant females, relative to subordinate females (and all males), express greater concentrations of A_4_ and E_2_, and greater or equivalent T concentrations (52). Unlike many cooperatively breeding species that experience physiological suppression of reproduction, adult meerkats of both sexes can reproduce throughout life (53). Indeed, within their natal clans, subordinate dams show normative glucocorticoid concentrations (53, 54) and produce offspring that survive in relevant numbers, some even ascending to the dominant position (27, 49, 54, 55). Nonetheless, female meerkats that express the highest androgen concentrations are, unusually, the most reproductively successful (51). These females are, however, also the most parasitized (56) and immunocompromised (57), suggesting important, androgen-mediated trade-offs.

During pregnancies carried to term within the clan, the exceptionally high, albeit status-related, androgen concentrations of dams (52) provide a means for the epigenetic transmission to offspring of androgen-mediated traits. In a prior study of the reproductive endocrinology and behavior of dominant control (DC), subordinate control (SC), and dominant treated (DT) dams that received the antiandrogen flutamide throughout late gestation, we found that DC dams express their peak hormone concentrations during late gestation (27), when feeding competition, dominance interactions, and evictions are magnified. They also produce offspring that are more aggressive in early postnatal development than those of SC dams, indicating normative maternal effects on infant behavior. Moreover, blocking androgen receptors in DT dams reduces their dominance interactions and is associated with infrequent evictions and decreased social centrality within the clan, as well as increased aggression in cohabiting SC dams. Lastly, flutamide treatment also reduces offspring aggression, confirming that maternal effects on infant behavior owe specifically to late-term androgens (27).

Although we lack information on fetal intrauterine position, key to examining fraternal effects (16, 17), we can largely dismiss the influence of fraternal androgens on status-related differences in meerkats. Firstly, masculinization of genitalia, as would occur either from mid-gestational maternal or fraternal androgens, is not present in meerkats, diminishing the likelihood of earlier androgen exposure from either source. Secondly, although meerkats produce mixed-sex litters [a proxy of fraternal androgen exposure in field studies (18)], maternal status does not affect the relatively even offspring sex ratios observed at first capture (58). These are also estimates of fetal offspring sex ratios – given that meerkats are born underground and can differentially fall victim to early neonatal mortality, infanticide, and predation, all prior to emergence – but even with greater pup mortality in subordinate matrilines, any discordance between pre- and post-birth sex ratios should be randomly distributed. Accordingly, differences in pup aggressive behavior by maternal status and treatment owe to maternal, rather than fraternal, androgens. Here, therefore, we test for further maternal effects, specifically, and for potential trade-offs in the offspring of the three ‘treatment’ groups of females (i.e., DC or high-T, SC or lower-T, and DT or blocked-T dams). Specifically, we examine developmental patterns in the same reproductive steroids (A_4_, T, and E_2_), as well as IGF-1, growth, and survivorship in the first year of life.

In species characterized by matrilineal dominance hierarchies, socialization effects can confound the relations between prenatal endocrine milieu and postnatal behavior or somatic development. For instance, maternally acquired dominance status across species can impact offspring aggression (59), mediate differences in food access (8), and affect IGF-1 concentrations and its consequences (41, 43, 45). Nevertheless, patterns observed in ‘normative’ species may not hold in masculinized females that are similarly characterized by maternal rank acquisition. In spotted hyenas, for instance, maternal rank (which predicts androgen-mediated offspring aggression: 24) positively influences female juvenile size independently of IGF-1, and high IGF-1 concentrations negatively impact survival in females after reproductive maturity (60). Teasing apart the social and physiological determinants of maternal effects can be challenging. Studies in the meerkat allow us to address this challenge because intense allocare minimizes maternally determined socialization effects (61) and, relative to non-cooperatively breeding species, allows better isolation of prenatal neuroendocrine effects on development (27).

Wild, yet habituated, meerkats represent an excellent study system that serves as both a ‘natural experiment’ of normative variation and allows ‘phenotypic engineering via hormonal manipulation’ [see (47)] in the field. This combined approach uniquely allows us to address the following main predictions. Based on reproductive endocrine patterns in adult meerkats (52) and developing spotted hyenas (36), we predict (i) raised A_4_ and minimal sex differences in the reproductive hormones of normative, developing offspring, including at puberty. Regarding treatment effects on reproductive hormones and other metrics, we expect (ii) the strength or rate of effects to vary by treatment group, particularly in an androgen-dependent fashion (DC > SC > DT). Regarding IGF-1 and growth, based on studies of various species experiencing raised androgen concentrations prenatally (5, 60, 62), we expect (iii) either a lack of sex difference in IGF-1 and growth or a reversed sex difference, with increased IGF-1 and growth in normative female meerkats. We also anticipate the typical age-related patterns, including a decline in IGF-1, but steady gain in growth. Lastly, based on the health costs of androgens (specifically increased parasitism and reduced immunocompetence) in female meerkats (56, 57) and their offspring (63), we predict (iv) an early survival cost in normative offspring – one that would be alleviated in flutamide-exposed animals. Because our study occurred during a prolonged drought, we also explore the socioecological conditions, including clan size (indicative of the number of helpers) and rainfall (indicative of the abundance of insects) [insects are the primary prey of meerkats (64)], that could have contributory effects on these various metrics. Our overall aim is thus to assess the degree to which androgen exposure is evolutionarily selected, in a manner that should contribute to our understanding of life-history theory and the trade-offs associated with routine female masculinization.

## Materials and methods

### Subjects and study site

Our focal subjects were 109 wild meerkats (*n* = 57 male, *n* = 52 female; **Table 1**) that were young members of 17 clans (ranging in size from 3-36 individuals) inhabiting the Kuruman River Reserve (26°58′S, 29°49′E), South Africa, during the study period 2012-2015. These young individuals derived from healthy DC, SC, and DT dams (**Table 1**), whose flutamide treatment regimens had been previously validated (65) and reported (27). Briefly, while under anesthesia (see sampling procedures outlined below), dominant females in late-gestation received flutamide (~15 mg/kg/day) via two, 21-day release pellets (300 mg, Innovative Research of America, Sarasota, FL), implanted subcutaneously by a licensed veterinarian. The treated animals also received a subcutaneous injection of a non-steroidal, anti-inflammatory painkiller (either 0.2–0.3 mg/kg meloxicam, Metacam, Boehringer or 0.1 ml of metacam, plus 0.1 ml of lentrax or other long-lasting penicillin, depending on availability). Dam identity was known and confirmed through genetics (66).

**Table 1:** Sample sizes by maternal treatment and offspring sex.

Maternal treatment	# Dams	# Litters	# Offspring (male; female)*	# Serum samples (male; female)**
Dominant control (DC)	13	15	50 (27; 23)	134 (66; 68)
Subordinate control (SC)	14	15	42 (23; 21)	109 (64; 45)
Dominant treated (DT)	5	5	15 (7; 8)	65 (34; 31)
Total	31	35	109 (57; 52)	308 (164; 144)

*Numbers reported are for the start of the study.

**Numbers reported are for the total number of samples available for endocrine analyses.

All individuals in this study were microchipped, habituated to human observers and to voluntary weighing, and were individually recognizable via dye-marks, as previously described (67). We monitored these offspring roughly three times per week, from pup emergence (at approximately 21-30 days of age) to adulthood (at 1 year) [see (68)]. We recorded the weights and life-history metrics of the subjects up to three times per observation day. We also captured, measured, and obtained a blood sample from each focal subject at 1, 3, 6, 9, and 12 months of age, as described below. Because subadult meerkats do not disperse (69, 70), individuals < 1 year of age that disappear from the clan are presumed dead; in such cases, we assigned the date of the last sighting as the date of death. Beyond the offspring’s metrics, we calculated its clan size by averaging daily values from the week preceding its capture. We obtained daily rainfall data from an onsite weather station (CR200 datalogger; Campbell Scientific) and used, for our analyses, cumulative rainfall from the month preceding capture.

### Sampling procedures

At ~ 1 month of age (range 18-36 days), we captured and sexed the focal subjects shortly after emergence and implanted permanent Passive Integrated Transponders or PIT tags. Because the fur of pups grows quickly, we individually marked 1-month-old pups by trimming small sections of their fur rather than by using hair dye. Next, we measured right hindfoot length (mm) using digital calipers. We then obtained tissue samples for genetic analysis by cutting a small piece (2 mm) from the tail tip with sterilized scissors. Following this procedure, we collected blood samples from the tail tip using heparinized capillary tubes.

At the older ages sampled, we individually captured and processed the focal subjects in the morning and at the den to minimize the time delay (mean ± S.E.M. = 8.30 ± 0.20 min) until blood draw (52). We gently picked up the subjects, by the tail base, carefully placed them into a cotton sack and anesthetized them with isoflurane (Isofor; Safe Line Pharmaceuticals, Johannesburg, South Africa) in oxygen, using a vehicle-mounted vaporizer (71). First, we drew an age-appropriate volume of 0.2–2 ml of blood from the jugular vein, using a 25-G needle and syringe. We allowed blood samples to clot at ambient temperature in serum separator tubes (Vacutainer^®^, Becton Dickinson, Franklin Lakes, NJ, USA) and later centrifuged them at 3700 rpm, at 24 °C, for 10 min. We stored decanted serum samples on site at −20 °C until transport, on ice, to Duke University in North Carolina, where they were stored at −80 °C until assay.

### Enzyme immunoassays

We assayed serum samples for A_4_, T, E_2_, and IGF-1 using commercial, competitive enzyme immunoassay (EIA) kits (ALPCO diagnostics, Salem, NH, USA). For all assays, we diluted with assay buffer, to a maximum of 1:8, any samples that had concentrations greater than the upper detection limit and multiplied the results obtained by the dilution factor. For samples with concentrations below the minimum detectable limit of the assay, we allocated the minimum assay value. We ran all samples in duplicate and, if the coefficient of variation (CV) exceeded 10%, we ran a subsequent assay. Capture-to-bleed time was recorded for nearly all (save *n* = 6) samples and found to be non-significant for A_4_, T, and E_2_ (ANOVA: *p* = 0.11, 0.56, and 0.31, respectively) but significant for IGF-1 (ANOVA: *p* = 0.01), with concentrations being weakly, but positively, related to the time delay to sample collection. Nevertheless, adding capture-to-bleed time neither improved the model fit nor altered the results, therefore we removed this variable from subsequent analyses (see below).

EIA serum assays of A_4_, T, and E_2_ have been previously validated in meerkats by standard parallelism, linearity, and recovery tests (52). The serum A_4_ assay has a sensitivity of 0.04 ng/ml using a 25-µl dose, with intra- and inter-assay CVs of 5.23% and 8.7%, respectively. The serum T assay has a sensitivity of 0.02 ng/ml using a 50-µl dose, with intra- and inter-assay CVs of 7.9% and 7.3%, respectively. The E_2_ assay has a sensitivity of 10 pg/ml using a 50-μl dose, with intra- and inter-assay CVs of 7.7% and 8.7%, respectively. Owing to smaller serum volumes obtained at younger ages, we lacked sufficient representation for meerkats at 1 month and therefore excluded this age from the analyses of E_2_.

The IGF-1 assay has a sensitivity of 0.09 ng/mL using a 10-μl dose, with intra- and inter-assay CVs of 7.7% and 8.7%, respectively. Serial dilutions of pooled meerkat serum yielded a displacement curve parallel to the IGF-1 standard curve. Assay accuracy, measured as percent recovery of known amounts of analyte from a pooled serum sample was 102.7% (*n* = 6). This assay has an initial step to dissociate IGF-1 from its binding proteins by dilution in an acidic buffer; following this step, there was no cross reactivity with IGF-II, insulin, or C-peptide.

### Statistical analysis

#### Subject considerations

The second cohort of offspring (in 2013) experienced severe drought. There were no litters from treated dominant dams in this cohort and drought conditions resulted in extremely low survivorship (87% mortality compared to an average of 40% for cohorts one and three). Nevertheless, including or excluding this year had no impact on endocrine results, so we included individuals from this year in all hormone analyses (see below). Owing to the death of some dominant animals during the span of the study, three of our subjects acquired dominance at an unusually young age. Because of the physiological and morphological changes associated with dominance acquisition (74), we excluded these subjects’ post-ascension blood samples (*n* = 5) and weight measures from our analyses. 

#### Generalized Additive Model

For each hormone, we analyzed developmental trajectories of serum concentrations using a Generalized Additive Model (GAM) with gamma error distributions, a log link function, and REML penalty, using the package *mgcv* 1.9-0 (72) in R version 4.3.2 (73). We used additive models to account for possible nonlinear relationships across development between hormone concentrations and continuous predictors, such as age. Maternal treatment (DC/SC/DT) and offspring sex (male/female) were included as fixed factors in the model. Because offspring weight was highly correlated with offspring age, we calculated size-corrected mass as a proxy of offspring body condition (i.e., separate from age-related size differences), using the residuals from two linear models (one per sex) wherein we regressed mass (g) with hindfoot length (mm) to correct for age-related structural size differences between individuals. We then included these residuals, as well as age in months, average clan size, and monthly rainfall as smooth terms in the model. We removed terms that were not significant and/or explained < 1% of the variation. If the empirical distribution function (EDF) was 1.000 (across all levels of factor), we removed smooths and instead included these terms as linear predictors. We included individual identity and litter as random effects to account for repeat sampling. We also initially included the interactions between sex and maternal treatment, as well as between sex and rainfall, in case the sexes responded differently to maternal treatment or environmental conditions. To enable interpretation of first-order effects, we dropped interactions if they were not significant and/or explained < 1% of the deviation.

Model selection was performed for the age smooth term using the lowest Akaike’s Information Criterion (AIC) score [at least 2 from the next best model; (75)] to determine which factors to include as “by” levels for this term: sex, maternal treatment, neither or both. All best models were at least 2 from the next best model except A_4_, for which another best fit model contained both “by” levels but the age by treatment (non-significant) term had an EDF approaching 0, indicating that this term contributed < 1 degree of freedom to the model. Due to REML penalization, which penalizes overfitting (76), this model was essentially equivalent to the other best model of age by sex alone. We thus report the simplified age-by-sex model. We confirmed that there were no high correlations (< 0.5) between independent terms and examined extreme residual values in quantile-quantile (QQ) plots of random effects to ensure that these values were randomly grouped and not accounted for by predictors. We used the DHARMa package (77) to confirm that there was no over- or under-dispersion, or residual spatial or temporal autocorrelation in the models, and we used the function ‘k.check’ in *mgcv* to optimize the number of knots (k value) in smooth terms. We plotted GAM model predictions on the response scale, using the base function predict and *ggplot2* 3.4.4 (78).

We also analyzed offspring weight change using GAMs, but with gaussian error distributions on the response scale. Model selection, diagnostics, plotting, and terms were the same as those used for hormone analyses, except that we included the average hindfoot length of offspring to account for skeletal size differences.

#### Generalized Linear Mixed Model

We modeled differences between offspring hormone concentrations and weight at 12 months (maturity) using a Generalized Linear Mixed Model (GLMM) with a gamma error structure (gaussian error for weight) and log link function, using the package *glmmTMB* 1.1.7 (79). We used the *DHARMa* package for GLMM model diagnostics and used the same terms as in the GAM (excluding age and individual).

We analyzed offspring survival using a GLMM with a binomial error structure and logit link function, using the package *glmmTMB*. We tested for collinearity between independent variables using variance inflation factors, ensuring an upper limit of three, and used the DHARMa package for GLMM model diagnostics. We included survival to the next breeding season as a binary response variable (0 for death, 1 for survival). We excluded individuals born in the second cohort (during 2013) to prevent any treatment-related survivorship bias. Additionally, we excluded individuals that had not been sexed prior to their last sighting (*n* = 16). We included dam treatment (DC/SC/DT), offspring sex (male/female), and both clan size and monthly rainfall at the time of pup emergence as fixed factors in the model; we included litter as a random factor.

## Results

**Figure 1:**
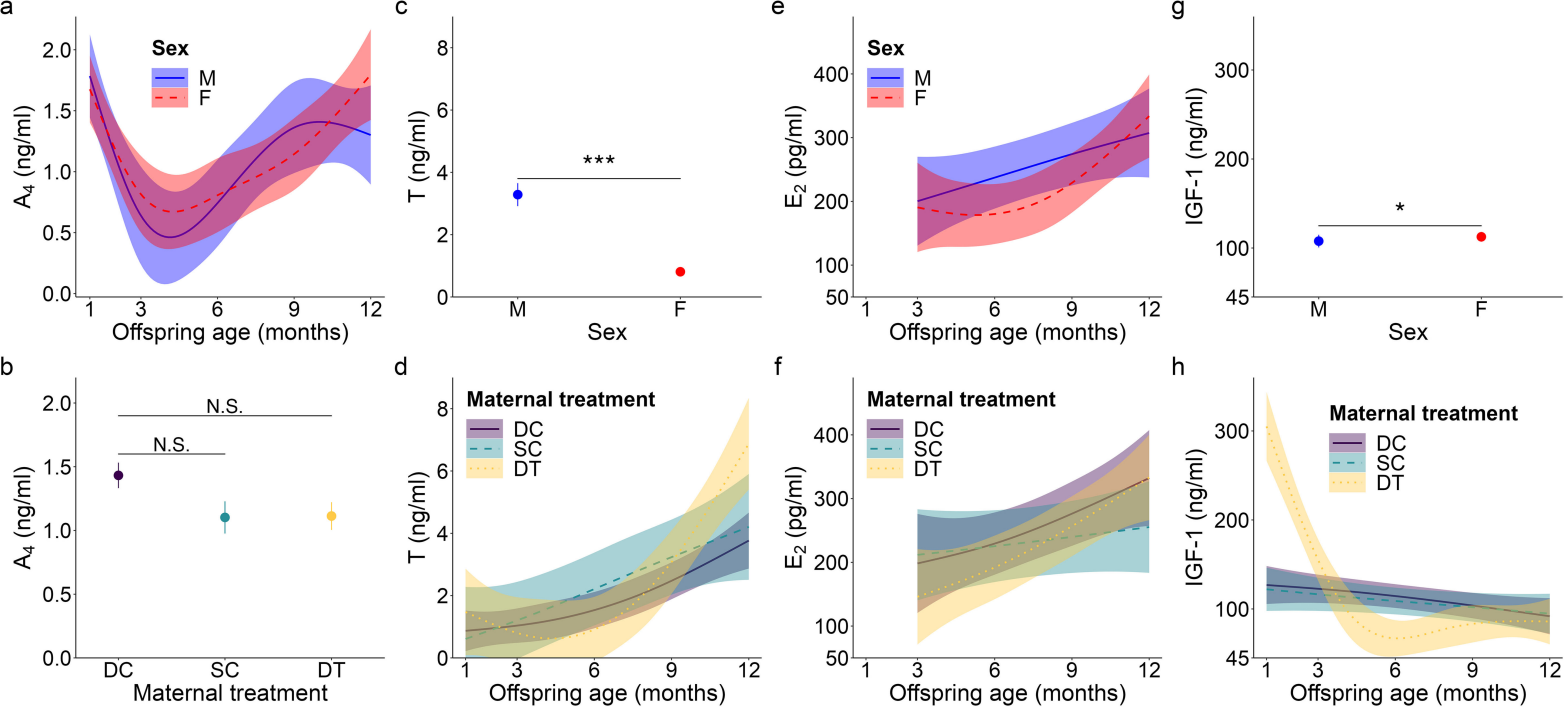
Top model-predicted serum concentrations of **(A, B)** androstenedione or A_4_, **(C, D)** testosterone or T, **(E, F)** estradiol or E_2_, and **(G, H)** insulin-like growth factor 1 or IGF-1 for meerkats by sex (male, M; female, F), age, and/or maternal treatment (dominant control, DC; subordinate control, SC; dominant treated, DT). For significant interactions between age and either sex **(A, E)** or maternal treatment **(D, F, H)**, the estimates are presented from 1-12 months with 95% CI. Otherwise, model predicted means ± s.e. are presented by sex **(C, G)** or maternal treatment **(B)**. Significance of comparison between groups is as follows: N.S. *p* > 0.05; * *p* < 0.05, *** *p* < 0.001.

**Figure 2:**
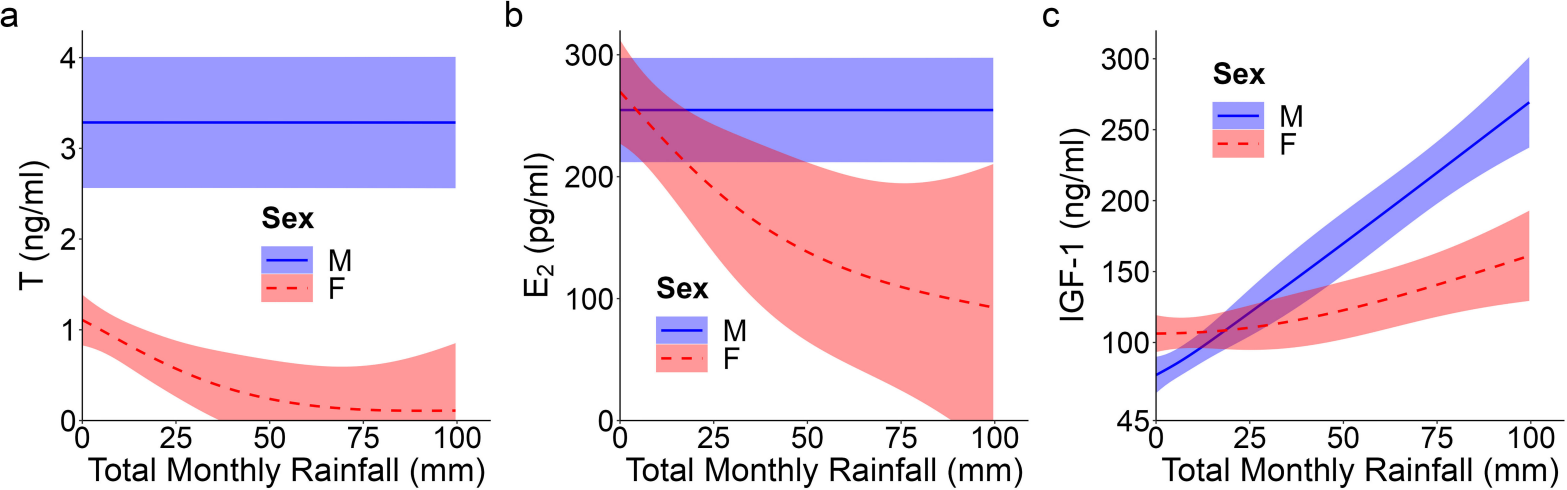
Top model-predicted serum concentrations of **(A)** testosterone or T, **(B)** estradiol or E_2_, and **(C)** insulin-like growth factor 1 or IGF-1 for meerkats by sex (male, M; female, F) and total monthly rainfall (mm). The estimates are presented with 95% CI.

**Figure 3:**
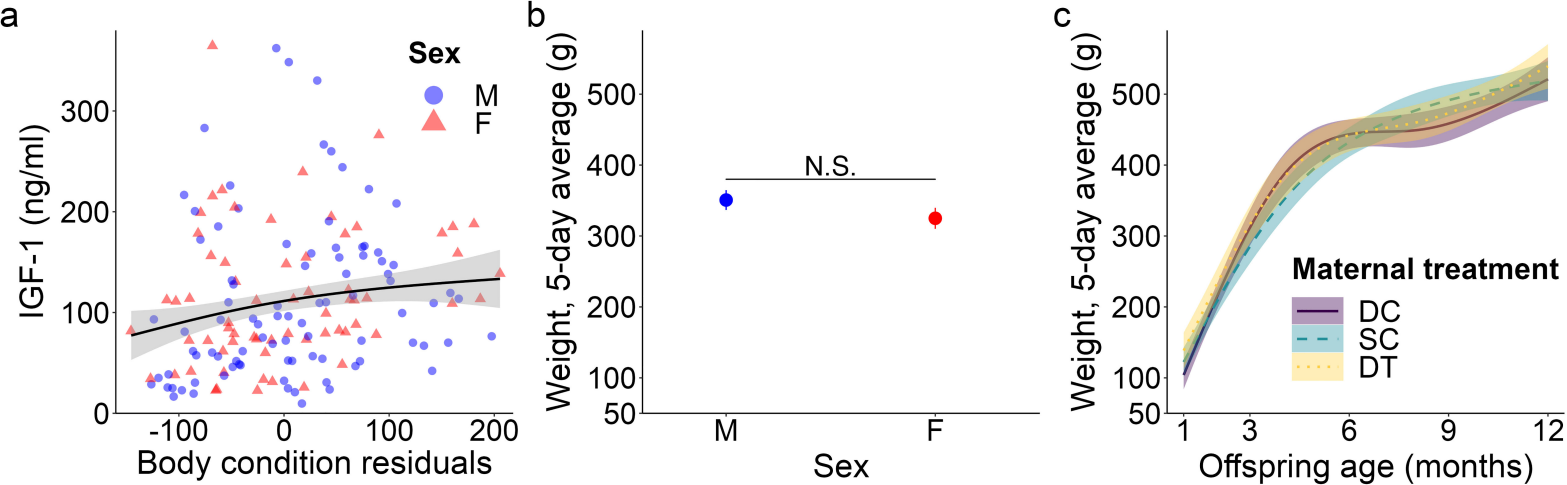
Top model-predicted **(A)** IGF-1 concentrations and **(B, C)** weights for meerkat offspring. In **(A)**, IGF-1 concentrations are predicted by body condition residuals. IGF-1 estimates are presented with their 95% confidence intervals (CI) and the raw data from 1-12 months are plotted by sex (male, M; female, F). In **(B)**, mean ± s.e. weights are predicted by sex, but are not significant (data thus represent all ages, 1-12 months). In **(C)**, weights are predicted by age and maternal treatment (dominant control, DC; subordinate control, SC; dominant treated, DT). For the significant interaction between age and maternal treatment, estimates are presented from 1-12 months with 95% CI, N.S. *p* > 0.05.

Our full results for the predictors of hormone concentrations (at all ages, **Supplementary Table S1**; at 12 months, **Supplementary Table S2**), offspring weight (at all ages, **Supplementary Table S3**; at 12 months, **Supplementary Table S4**), and survivorship (to 12 months, **Supplementary Table S5**) are presented in the **Supplementary Material**, with significant findings and data visualization highlighted below. For ease of visualization, **Figures 1**–**3** emphasize the model lines; respective **Supplementary Figures S1**-**S3** present the range of individual data points and **Supplementary Figure S4** presents hormone concentrations by body condition residuals.

### Androstenedione

For offspring of both sexes, A_4_ concentrations showed a quadratic age effect (GAM: females, *F* = 3.36, *p* = 0.019; males, *F* = 3.34, *p* = 0.017; for effect sizes/estimates, see **Supplementary Table S1**), with an atypical peak at 1 month, followed by a decrease, and then the more usual, gradual increase as juvenile meerkats approached puberty and adulthood (**Figure 1A**). Also unusual for most mammals, but consistent with prediction (i) based on masculinized species, was the lack of a main effect of sex in A_4_ concentrations, with female values being as high as male values, even at sexual maturity (GLMM: 12 months, χ^2^_1_ = 1.85, *p* = 0.064; **Supplementary Table S2**; **Figure 1A**). Despite a suggestive pattern, there was no significant effect of maternal treatment on A_4_ concentrations (**Figure 1B**), contrary to prediction (ii). We observed no notable patterns in A_4_ concentrations in relation to either clan size or rainfall. There was, however, a significant effect of body condition (GAM: *F* = 6.87, *p* < 0.001; **Supplementary Table S1**), with offspring at the ‘extremes’ of body condition (i.e., those at either end of the distribution) having reduced A_4_ concentrations relative to offspring in average body condition (**Supplementary Figure S4A**).

### Testosterone

Unlike patterns observed for A_4_, an initially typical sex effect emerged for T, with males having greater average concentrations than females throughout early development (GAM: female vs. male, *t* = -6.78, *p* < 0.001; **Supplementary Table S1**; **Figure 1C**); however, this difference no longer held at maturity, when female values matched those of males (GLMM: 12 months, χ^2^_1_ = -0.61, *p* = 0.542; **Supplementary Table S2**). Prediction (i) thus held for pubertal values. T concentrations in the offspring of DT dams showed a quadratic age effect (GAM: *F* = 3.50, *p* = 0.032; **Supplementary Table S1**), with an atypical peak at 1 month that was absent in the offspring of DC or SC dams (**Figure 1D**). Instead, the latter two groups of offspring showed a more consistent increase to puberty (**Figure 1D**). Overall, however, T concentrations did not differ by maternal treatment. Although there were effects of treatment, they did not occur in a linear, androgen-dependent fashion, as outlined in prediction (ii). There was also a significant influence of rainfall on the T concentrations of females (GAM: *t* = 3.84, p = 0.031; **Supplementary Table S1**): whereas male values remained consistent across wet and dry conditions, female values decreased with increasing rainfall (**Figure 2A**). Lastly, T concentrations (as with A_4_ concentrations), showed a quadratic effect of body condition (GAM: *F* = 28.04, *p* < 0.001; **Supplementary Table S1**), with reduced T concentrations at the extremes of body condition; **Supplementary Figure S4B**).

### Estradiol

As with A_4_, overall E_2_ concentrations showed no sex effect across the ages sampled, not even at maturity (GLMM: 12 months, χ^2^_1_ = 1.46, *p* = 0.145; **Supplementary Table S2**; **Figure 1E**), consistent with prediction (i). In females, there was a quadratic age effect (GAM: *F* = 8.99, *p* < 0.001; **Supplementary Table S1**), evidenced by a decrease in E_2_ after weaning (at 3 months) followed by a gradual increase in E_2_ from 6 months to puberty (**Figure 1E**). Males had a non-significant linear trend with age (GAM: *F* = 0.88, *p* = 0.351; **Supplementary Table S1**). We also observed a significant positive and linear age effect on E_2_ in the offspring of DT dams (GAM: *F* = 7.10, *p* = 0.009; **Supplementary Table S1**), but not in the offspring of DC or SC dams (**Figure 1F**). As with androgens, we detected no effect of maternal treatment on overall E_2_ concentrations. Again, although there were effects of treatment, they did not occur in the order outlined in prediction (ii). There was, however, a significant linear, sex-by-rainfall interaction on E_2_ concentrations (GAM: *t* = -3.03, *p* = 0.003), with male and female meerkats having opposing responses to this environmental variable: E_2_ was not associated with rainfall in males but was negatively associated in females (**Supplementary Table S1**; **Figure 2B**). Lastly, whereas clan size was negatively associated with E_2_ concentrations (GAM: *t* = -3.15, *p* = 0.002; **Supplementary Table S1**), offspring body condition positively related to E_2_ concentrations (GAM: *F* = 3.97, *p* = 0.048; **Supplementary Figure S4C**).

### Insulin-like growth factor-1

We detected a significant main effect of sex on IGF-1 concentrations (GAM: *t* = 2.31, *p* = 0.023; **Supplementary Table S1**), with female values exceeding male values, even at maturity (GLMM: 12 months, χ^2^_1_ = 1.58, *p* = 0.114; **Supplementary Table S2**; **Figure 1G**), consistent with prediction (iii). Age differentially predicted IGF-1 concentrations across maternal treatments (GAM: DC x age, *F* = 3.30, *p* = 0.040; SC x age, *F* = 3.04, *p* = 0.016; DT x age, *F* = 2.47, *p* = 0.069; **Supplementary Table S1**), in that the offspring of DC and SC dams showed steadily decreasing IGF-1 concentrations with age, whereas the offspring of DT dams showed initially higher IGF-1 concentrations at emergence, that then plummeted until 6 months and stabilized thereafter (**Figure 1H**). Nonetheless, average IGF-1 concentrations did not differ by maternal treatment. Again, treatment effects were more variable than those outlined in prediction (ii). Social and environmental factors also impacted IGF-1 (GAM: t = -2.81, *p* = 0.006; **Supplementary Table S1**), as offspring raised in larger clans had reduced IGF-1 compared to counterparts raised in smaller clans. Moreover, there was a significant, positive association between IGF-1 and total monthly rainfall (GAM: *t* = 4.75, *p* < 0.001; **Supplementary Table S1**), as well as a significant sex-by-rainfall interaction (GAM: *t* = -2.07, p = 0.041; **Supplementary Table S1**); in this case, whereas both sexes showed a positive relation between rainfall and IGF-1 concentrations, male meerkats were more sensitive than females to this environmental variable (**Figure 2C**). Lastly, as observed with E_2_, offspring in better body condition showed increased IGF-1 concentrations (GAM: t = 2.94, *p* = 0.004; **Supplementary Table S1**; **Figure 3A**).

### Body weight

We detected no significant sex difference in body weight across the ages sampled (**Figure 3B**), consistent with prediction (iii). Also as expected, offspring body weight increased with age; however, there was a significant influence of maternal treatment on the rate (i.e., slope) of weight gain (GAM: DC x age, *F* = 44.33, *p* < 0.001; SC x age, *F* = 67.67, *p* < 0.001; DT x age, *F* = 35.26, *p* < 0.001). Whereas offspring from SC dams showed relatively steady weight gain until 1 year, with a slower initial pace and earlier evidence of reaching the adult plateau, their counterparts from DC and DT dams showed more variability in pace, with initially faster weight gain, followed by a plateau between 6-9 months, and finally a second growth spurt as they approached maturity (**Figure 3C**). Ultimately, by 12 months of age, there was no weight difference between the three groups (GLMM: 12 months, SC vs. DC χ^22^ = -0.81, *p* = 0.419; DT vs. DC χ^22^ = -0.94, *p* = 0.347). Again, significant treatment effects were more variable than those outlined in prediction (ii). Clan size also significantly impacted body weight (GAM: *F* = 18.28, *p* < 0.001), with individuals in larger clans being heavier, except in exceptionally large clans (of > 30 individuals) wherein individuals were lighter. Lastly, increased rainfall was not significantly associated with body weight (GAM: *F* = -1.68, *p* = 0.094); rainfall still was not a significant predictor of weight at a given age class.

### Survivorship

**Figure 4:**
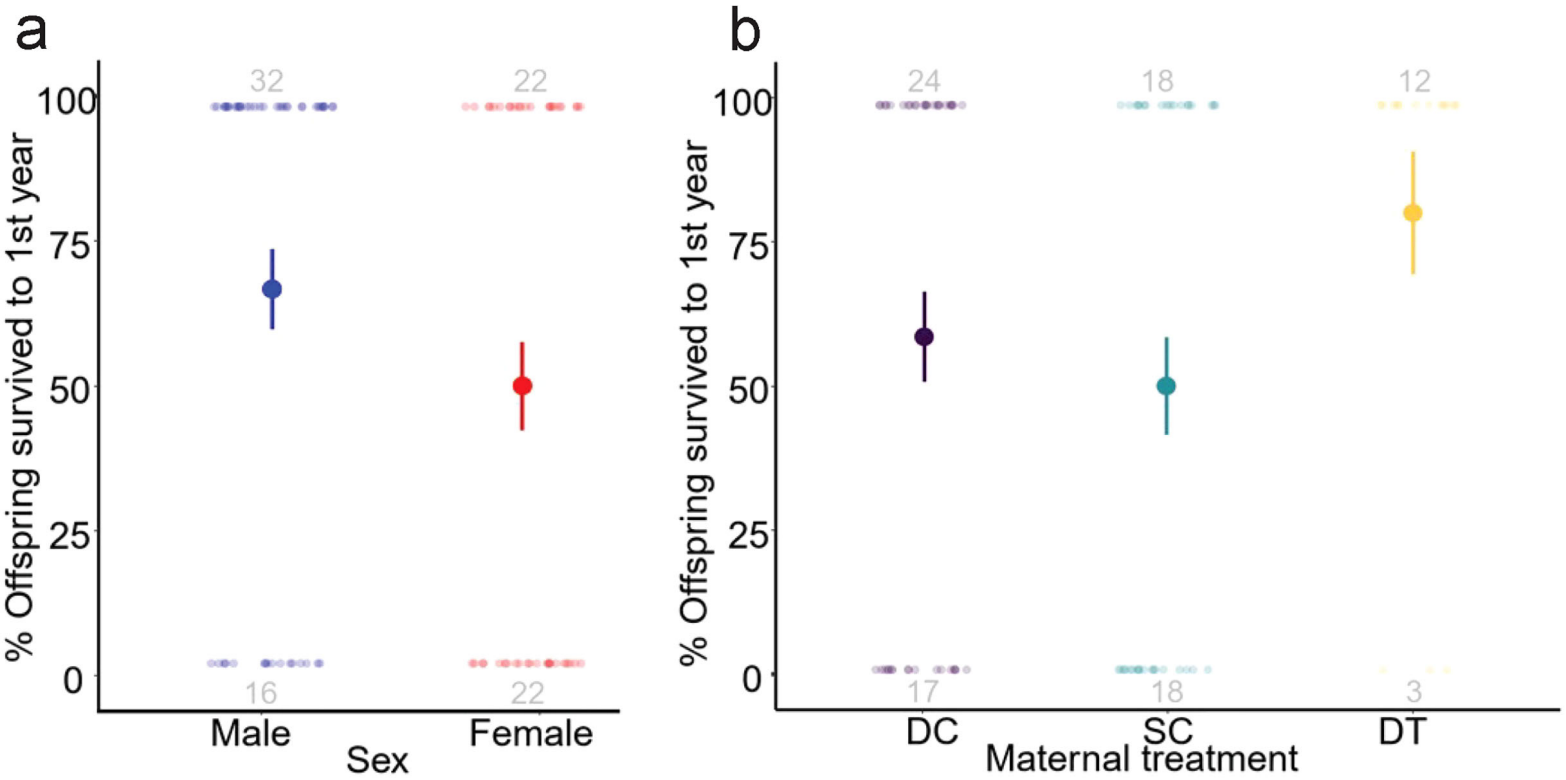
Likelihood (%) survival of meerkat offpsring to one year of age by **(A)** sex and **(B)** maternal treatment (dominant control, DC; subordinate control, SC; dominant treated, DT). Data presented are raw means ± s.e. with sample sizes of individuals surviving to one year (top) and dying before one year (bottom) included in grey.

During our period of study, young male meerkats were more likely to survive to adulthood than were young females (GLMM: z = 2.01, *p* = 0.044; **Figure 4A**). There was no significant association between maternal treatment and the probability of surviving to adulthood; however, offspring born to DT dams tended to have increased survival likelihood (GLMM: *p* = 0.095; **Figure 4B**), partially consistent with prediction (iv). Notably, 80% of the offspring born to DT dams survived to one year, compared to only 59% and 50% of the offspring born to DC and SC dams, respectively. There was also a slight, but positive, association between total monthly rainfall at emergence and survivorship (GLMM: z = -3.01*, p* = 0.003).

## Discussion

As a test of maternal endocrine regulation of offspring developmental trajectories in a wild mammalian population, our results show the importance of longitudinal, experimental studies in nature for understanding life-history trade-offs [for a related discussion, see (41)]. From the time meerkat pups emerged from their subterranean den to the time they reached adulthood, we observed variable effects of offspring sex, age, and prenatal androgen exposure on both absolute metrics and rates of change, some of which were modulated by individual body condition, clan size, or prey abundance. Notably, although we may have lacked the resolution necessary to detect a linear correlation between androgen action (i.e. DC > SC > DT) and our various metrics, significant effects of maternal treatment on rates of T, E_2_, and IGF-1 expression, as well as on weight and potentially survivorship, confirm that the maternal endocrine environment impacts sexually differentiated, postnatal physiological and somatic development.

### Reproductive hormones

Despite the functional significance of estrogens in male vertebrates and androgens in female vertebrates (40, 80), studies of mammalian development that compare the same hormones across both sexes remain limited. Nonetheless, existing ‘sex-comparative’ studies [e.g (81)] show endocrine patterns that are consistent with ‘sex-specific’ studies [e.g (39)], generally showing low initial steroid concentrations in infants of both sexes, followed by a gradual, sometimes exponential, increase of androgens in males and of estrogens in females as animals approach puberty [reviewed in (82)]. Masculinization of females via fraternal or maternal androgens can, however, alter postnatal physiology, both in absolute hormone concentrations and in developmental patterns (17, 35, 36). Indeed, as anticipated in prediction (i), young meerkats showed departures from this generalized mammalian pattern of development, further illustrating the meerkat’s unusual physiological path to cooperative breeding (27, 53).

Young meerkats showed an initial male-bias in T that disappeared by one year of age, generally consistent with the unusual pattern observed in adult subordinates (52). By contrast, A_4_ and E_2_ concentrations were equivalent in both sexes and greater than expected at young ages, again comparable at one year to those expressed by older meerkats not actively engaged in reproduction (52). By comparison, A_4_ concentrations in young, female spotted hyenas exceed those of males and older juvenile and nonpregnant adult females (35, 36). In both masculinized species, androgen production in females is enhanced from an early age. Meerkats from all three maternal treatment groups showed a particularly uncharacteristic peak in A_4_ at one month of age, inconsistent with their different hormonal milieus during the late prenatal period (27). The time frame from birth to 1 month coincides with lactation, which offers another avenue for maternal regulation of offspring development (83), including via transfer of steroid hormones and growth factors (84). Androgens in milk generally occur in low concentrations, but during bovine pregnancy, for example, values of A_4_ in milk are twice those in plasma, suggesting that the mammary gland either uptakes or synthesizes A_4_ (85). It is likely that raised A_4_ concentrations in meerkat pups owe to maternal transfer during nursing and reflects the adult females’ unusually high A_4_ production year-round [see (52)]. By comparison, maternal androgen concentrations in spotted hyenas plummet postpartum (86). Confirmation of androgen transfer in meerkats would require assessment of the HPG axis in nursing pups to exclude endogenous production. Nevertheless, the concordance between early pup behavior and late-gestational androgens in the three maternal treatment groups suggests that the organizational window for behavioral effects in meerkats closes shortly after birth (27). The impact of raised steroid concentrations in infancy requires further investigation in this and other species.

Likewise understudied is the role of E_2_ in males. The anticipated, yet uncharacteristically high E_2_ concentrations in male meerkats may owe to aromatization of androgens, potentially with functional relevance. In the blue-eyed black lemur (*Eulemur flavifrons*) — another female-dominant primate in which females display masculinized traits — E_2_ concentrations are raised in adult males (87). Additionally, in cooperatively breeding naked mole-rats (*Heterocephalus glaber*), E_2_ is raised in both sexes when the queen is pregnant (88). E_2_ in males has been shown to enhance nurturing paternal behavior (89, 90) and may be similarly linked to allocare in young male meerkats [i.e., beyond cortisol-mediated male care; (91)]; however, this potential functional explanation remains speculative.

Lastly, social and ecological influences on reproductive hormones are well known, particularly as regards rank-related and seasonal differences, evidenced, for example, by the Challenge Hypothesis (92) or during succession in cooperative breeders (93). In meerkats, clan size (94) and rainfall (95) profoundly impact life-history variables and, likewise, influenced physiological ontogeny. In terms of social influence, we detected a negative effect of clan size on E_2_ in both sexes. If E_2_ facilitates allocare, one might expect a reduction in E_2_ in larger clans that have more helpers lightening the per capita load of allocare [even if load distribution is not equitable; see (96)]. In terms of ecological influence, we detected a modulatory effect of rainfall on female T and E_2_ concentrations, suggesting that female reproductive physiology may be more sensitive than that of males to environmental/nutritional variables, consistent with the key role of female-female competition in meerkat society. When resources are plentiful, perhaps the reduced need for intrasexual female competition ‘relaxes’ or lowers steroid hormone concentrations in females.

### IGF-1 and growth

Consistent with the prior findings and relative to various other mammals [e.g., pigs, *Sus scrofa domesticus*: (97); mice, *Mus musculus*: (98); mandrills, *Mandrillus sphinx*: (45)], meerkats are also unusual in their ‘reverse’ sex difference in IGF-1, following prediction (iii). Prior to maturity, female meerkats had higher IGF-1 concentrations than did males, consistent with early development in the spotted hyena (25, 99), as well as in humans (100). The factors underlying increased IGF-1 in females vs. males remain unclear; however, exposure to sex steroids *in utero* may be partially responsible. Experimental increases in maternal T negatively impact IGF-1 bioavailability and fetal growth in female sheep [*Ovis aries*: (101)]; however, postnatally, masculinized female lambs show increased IGF-1 concentrations and growth — results that have been replicated across studies and taxa [sheep: (101, 102); cattle, *Bos taurus*: (103)]. Although increased IGF-1 and growth represent potential postnatal benefits of androgen exposure for naturally masculinized females, it remains unclear if experimentally induced effects of increased androgens in other species owe to the direct actions of androgens and/or involve aromatization to estrogens. In our study, experimentally blocking androgen action *in utero* did not impact overall IGF-1 concentrations but did impact relationships of IGF-1 with age, suggesting a complex combination of developmental influences on IGF-1. The differing rates of change in IGF-1 by maternal treatment may indicate that androgen exposure initially reduces IGF-1 concentrations, as seen prenatally and during the early postnatal period in sheep (101). In domestic dogs (*Canis familiaris*), intact males have reduced IGF-1 relative to neutered males, and intact males have more gradual declines in IGF-1 (104), like the age patterns we observed in DC vs. DT offspring. Surprisingly, we did not detect a relationship between rainfall and weight, potentially owing to a floor effect of low rainfall (see **Supplementary Figure S2**). Alternately, because pups emerged from their dens during the periods of highest rainfall, the lowest weights of these young animals may have obscured any relationship with rainfall. This null result may, however, suggest that the effects of IGF-1 and/or androgens in this study were independent of ecological variables.

Body condition is commonly understood to relate positively with IGF-1 concentrations (105): IGF-1 promotes healthy skeletal development and weight gain (106) and is reduced in early life by poor nutrition (107). Our findings may also be consistent with some nutritional dependency of IGF-1, particularly during a drought. Although we generally found offspring weight to increase with clan size or the increasing number of helpers available to feed offspring, consistent with a prior report (108), this pattern did not hold for clans of > 30 members. At extreme clan sizes (which were not included in earlier work), offspring weights were reduced, consistent with a clan size vs. IGF-1/growth trade-off, potentially driven by increased food competition. We also note a negative relationship with clan size and IGF-1, possibly indicative of larger clans experiencing some nutritional stress or harsher internal competition for nutrients that reduce IGF-1 concentrations via glucocorticoids (109, 110). The deviation from expected patterns for IGF-1 and clan size parallels other systems wherein variable nutrition weakens or eliminates positive associations between IGF and weight gain [reviewed in (111)].

We also note a modulatory role of offspring sex on the positive response of IGF-1 concentrations to rainfall, suggesting that males may be more sensitive to resource-driven increases in IGF-1 concentrations than are females. Even in the face of increased resource availability, competition may be a factor limiting nutrition and growth promotion (or IGF-1) in females. Although we observed no difference in growth rate based on offspring sex, maternal status was predictive of growth patterns: offspring from dominant dams showed increased growth in early life before all offspring equalized in body weight upon reaching adulthood. Positive associations between prenatal androgen exposure and postnatally accelerated growth rates have been shown in several taxa [e.g (5, 60, 62, 101, 112)]. Although androgen-accelerated growth rate can temporarily benefit offspring, by allowing them to out-compete rivals, it can also result in trade-offs that may negate derived benefits associated with maternal status. For example, differences in maternal status can be associated with differences in telomere length (113) or immune response (5, 57, 114–116), which can, in turn, negatively impact survival.

### Survivorship

Our finding of increased survival of male, relative to female, offspring during the period from emergence to adulthood contrasts with prior studies on the same population, in which there was either no difference in survival between the sexes from emergence or independence to adulthood (108, 113) or female offspring were more likely to survive between emergence and independence (61). This variation likely owes to fluctuations in key variables that were captured during long-term study of this population and provides a cautionary note when considering life-history characterizations based on shorter-term, single studies. Survivorship differences in relation to maternal status also vary for this same population, with prior researchers reporting both increased (61) and decreased (113) survival of offspring deriving from DC versus SC dams. Although we found no statistical difference in offspring survival to one year based on maternal treatment, offspring born to DT dams, that experienced androgen receptor blockade, tended to have increased survival relative to either set of normative dams, partially consistent with prediction (iv) – a pattern that continued after this study’s one-year cutoff (data not presented). Normative dominant and subordinate female meerkats express different androgen concentrations, but their developing offspring nonetheless experience higher androgen concentrations than is typical of most species. In this regard, the potential difference in survival between offspring that experienced androgens in late gestation versus those that did not is broadly consistent with the negative effects of maternally derived androgens on offspring survival reported in other species (4, 5, 116, 117).

Variability in survival within our population could notably owe to changing climatic conditions or health threats [including increased tuberculosis-related deaths; (118)] across years. Our study was conducted during a period of low rainfall and increased temperature (54) — environmental factors that have been shown to reduce meerkat pup survival and growth over time, particularly in smaller clans (119, 120). Indeed, we saw 48% mortality in pups, whereas in a prior analysis, pups showed only 11% mortality (61). Under such dire conditions, maintaining clan size may require subordinate reproduction. For the dams that contributed pups to our study, maternal status-related disparity in reproductive failure was also reduced (54), such that equal, total numbers of pups were born to DC and SC dams. This reduction in reproductive skew could have contributed to eliminating survival differences between DC and SC offspring. Drought conditions can also enhance maternally derived survival through differences in food acquisition and growth, for example via increased early life aggression in DC compared to SC offspring (27). Despite relatively small samples sizes, our interpretation that DT offspring are generally protected from the detrimental effects of androgens on survival is substantiated by evidence showing that prenatal blockade of androgen receptors also reduces parasitism and improves pup immunocompetence relative to DC offspring (63).

### Conclusion

As an exceptional species in which females variably experience raised androgen concentrations throughout life, the meerkat sheds new light on maternal effects and their associated trade-offs, illustrating the benefits of broadening our research coverage to non-model organisms. Androgen-mediated, competitive benefits starting in infancy and reproductive success accrued in adulthood may be associated with some age- and sex-related trade-offs experienced early in life. We suggest that the costs of androgens in meerkats, particularly in dominant matrilines and with reference to health and survival, may be most evident during ecologically challenging periods (54). Here, we best observed this effect through the seemingly enhanced survival of DT offspring. Although our findings in meerkats share similarities with other exceptional cases of androgen ‘excess’ in females (23, 121–123), they generally differ from those in other cooperatively breeding species (53), as well as from ‘normative’ females that experience experimentally raised androgen concentrations prenatally [e.g (124)]. Together, such differences call for greater understanding of the role of natural endocrine variation, across hormones, in both sexes. From a health perspective, we still have much to gain from these ‘experiments of nature,’ particularly as regards understanding the mechanisms and pathways that differentially modulate androgenic action between the sexes [including, for example, sex differences in gonadal ontogeny, the natural antiandrogenic action of epitestosterone, and receptor sensitivity or distribution (40, 125, 126)], or that protect against the reproductively negative consequences of ‘excess’ androgen exposure in other species, including our own (127–129).

## Data Availability

The datasets presented in this study can be found in online repositories. The names of the repository/repositories and accession number(s) can be found below: https://github.com/cls83211/davies-sheareretal2024.

## References

[1] Wilson JD. Sexual differentiation. Annu Rev Physiol. (1978) 40:279–306. doi: 10.1146/annurev.ph.40.030178.001431 345951

[2] Arnold AP. A general theory of sexual differentiation. J Neurosci Res. (2017) 95:291–300. doi: 10.1002/jnr.23884 27870435 PMC5369239

[3] Bernardo J. Maternal effects in animal ecology. Am Zoologist. (1996) 36:83–105. doi: 10.1093/icb/36.2.83

[4] Sockman KW Schwabl H. Yolk androgens reduce offspring survival. Proc R Soc London Ser B: Biol Sci. (2000) 267:1451–6. doi: 10.1098/rspb.2000.1163 PMC1690699 10983830

[5] Navara KJ Hill GE Mendonça MT. Variable effects of yolk androgens on growth, survival, and immunity in eastern bluebird nestlings. Physiol Biochem Zool. (2005) 78:570–8. doi: 10.1086/430689 15957111

[6] Sheriff MJ Bell A Boonstra R Dantzer B Lavergne SG McGhee KE. Integrating ecological and evolutionary context in the study of maternal stress. Integr Comp Biol. (2017) 57:437–49. doi: 10.1093/icb/icx105 PMC5886325 28957523

[7] Edwards PD Lavergne SG McCaw LK Wijenayake S Boonstra R McGowan PO. Maternal effects in mammals: broadening our understanding of offspring programming. Front Neuroendocrinol. (2021) 62:100924. doi: 10.1016/j.yfrne.2021.100924 33992652

[8] Altmann J Alberts SC. Growth rates in a wild primate population: ecological influences and maternal effects. Behav Ecol Sociobiol. (2005) 57:490–501. doi: 10.1007/s00265-004-0870-x

[9] Kaiser S Sachser N. The effects of prenatal social stress on behaviour: mechanisms and function. Neurosci Biobehav Rev. (2005) 29:283–94. doi: 10.1016/j.neubiorev.2004.09.015 15811499

[10] Goy RW Bercovitch FB McBrair MC. Behavioral masculinization is independent of genital masculinization in prenatally androgenized female rhesus macaques. Hormones Behavior. (1988) 22:552–71. doi: 10.1016/0018-506X(88)90058-X 3235069

[11] Clemens LG Gladue BA Coniglio LP. Prenatal endogenous androgenic influences on masculine sexual behavior and genital morphology in male and female rats. Hormones Behavior. (1978) 10:40–53. doi: 10.1016/0018-506X(78)90023-5 658890

[12] vom Saal FS. Sexual differentiation in litter-bearing mammals: influence of sex of adjacent fetuses in utero. J Anim Sci. (1989) 67:1824–40. doi: 10.2527/jas1989.6771824x 2670873

[13] Bánszegi O Altbäcker V Bilkó Á. Intrauterine position influences anatomy and behavior in domestic rabbits. Physiol Behavior. (2009) 98:258–62. doi: 10.1016/j.physbeh.2009.05.016 19490922

[14] Clark MM Galef BG. Prenatal influences on reproductive life history strategies. Trends Ecol Evolution. (1995) 10:151–3. doi: 10.1016/S0169-5347(00)89025-4 21236985

[15] vom Saal FS Bronson FH. Sexual characteristics of adult female mice are correlated with their blood testosterone levels during prenatal development. Science. (1980) 208:597–9. doi: 10.1126/science.7367881 7367881

[16] Vandenbergh JG Huggett CL. The anogenital distance index, a predictor of the intrauterine position effects on reproduction in female house mice. Lab Anim Sci. (1995) 45:567–73. 8569159

[17] Ryan BC Vandenbergh JG. Intrauterine position effects. Neurosci Biobehav Rev. (2002) 26:665–78. doi: 10.1016/S0149-7634(02)00038-6 12479841

[18] Monclús R Blumstein DT. Litter sex composition affects life-history traits in yellow-bellied marmots. J Anim Ecol. (2012) 81:80–6. doi: 10.1111/j.1365-2656.2011.01888.x 21801175

[19] McFadden D. A masculinizing effect on the auditory systems of human females having male co-twins. Proc Natl Acad Sci. (1993) 90:11900–4. doi: 10.1073/pnas.90.24.11900 PMC48092 8265645

[20] Bütikofer A Figlio DN Karbownik K Kuzawa CW Salvanes KG. Evidence that prenatal testosterone transfer from male twins reduces the fertility and socioeconomic success of their female co-twins. Proc Natl Acad Sci. (2019) 116:6749–53. doi: 10.1073/pnas.1812786116 PMC6452670 30886089

[21] Zielinski WJ vom Saal FS Vandenbergh JG. The effect of intrauterine position on the survival, reproduction and home range size of female house mice (*Mus musculus*). Behav Ecol Sociobiol. (1992) 30:185–91. doi: 10.1007/BF00166702

[22] Correa LA Leon C Ramírez-Estrada J Soto-Gamboa M Sepulveda RD Ebensperger LA. Masculinized females produce heavier offspring in a group living rodent. J Anim Ecol. (2016) 85:1552–62. doi: 10.1111/1365-2656.12588 27589255

[23] Drea CM Weldele ML Forger NG Coscia EM Frank LG Licht P. Androgens and masculinization of genitalia in the spotted hyaena (*Crocuta crocuta*). Effects Prenatal Anti-androgens Reproduction. (1998) 113:117–27. doi: 10.1530/jrf.0.1130117 9713384

[24] Dloniak SM French JA Holekamp KE. Rank-related maternal effects of androgens on behaviour in wild spotted hyaenas. Nature. (2006) 440:1190–3. doi: 10.1038/nature04540 16641996

[25] Conley A Place NJ Legacki EL Hammond GL Cunha GR Drea CM. Spotted hyaenas and the sexual spectrum: reproductive endocrinology and development. J Endocrinol. (2020) 247:R27–44. doi: 10.1016/j.tem.2006.09.005 32755997

[26] Grebe NM Fitzpatrick C Sharrock K Starling A Drea CM. Organizational and activational androgens, lemur social play, and the ontogeny of female dominance. Hormones Behavior. (2019) :115:104554. doi: 10.1016/j.yhbeh.2019.07.002 31276664

[27] Drea CM Davies CS Greene LK Mitchell J Blondel DV Shearer CL. An intergenerational androgenic mechanism of female intrasexual competition in the cooperatively breeding meerkat. Nat Commun. (2021) 12:7332. doi: 10.1038/s41467-021-27496-x 34921140 PMC8683399

[28] Jost A. Recherches sur la differenciation sexuelle de l’embrion de lapin. III. Role des gonades foetales dans la differenciation sexuelle somatique. Arch d’Anatomie Microscopique Morphol Experimentale. (1947) 36:271–315.

[29] Jost A. Hormonal factors in the sex differentiation of the mammalian foetus. Philos Trans R Soc London B Biol Sci. (1970) 259:119–31. https://www.jstor.org/stable/2417046. 10.1098/rstb.1970.0052 4399057

[30] Phoenix CH Goy RW Gerall AA Young WC. Organizing action of prenatally administered testosterone propionate on the tissues mediating mating behavior in the female Guinea pig. Endocrinology. (1959) 65:369–82. doi: 10.1210/endo-65-3-369 14432658

[31] Compston JE. Sex steroids and bone. Physiol Rev. (2001) 81:419–47. doi: 10.1152/physrev.2001.81.1.419 11152762

[32] Callewaert F Boonen S Vanderschueren D. Sex steroids and the male skeleton: a tale of two hormones. Trends Endocrinol Metab. (2010) 21:89–95. doi: 10.1016/j.tem.2009.09.002 19837603

[33] Birzniece V Ho KK. Sex steroids and the GH axis: implications for the management of hypopituitarism. Best Pract Res Clin Endocrinol Metab. (2017) 31:59–69. doi: 10.1016/j.beem.2017.03.003 28477733

[34] Shaw G Renfree MB Short RV. Primary genetic-control of sexual-differentiation in marsupials. Aust J Zool. (1989) 37:443–50. doi: 10.1071/ZO9890443

[35] Glickman SE Frank LG Davidson JM Smith ER Siiteri PK. Androstenedione may organize or activate sex-reversed traits in female spotted hyenas. Proc Natl Acad Sci. (1987) 84:3444–7. doi: 10.1073/pnas.84.10.3444 PMC304887 3472215

[36] Glickman SE Frank LG Pavgi S Licht P. Hormonal correlates of ‘masculinization’ in female spotted hyaenas (*Crocuta crocuta*). 1. Infancy to sexual maturity. Reproduction. (1992) 95:451–62. doi: 10.1530/jrf.0.0950451 1518001

[37] Yalcinkaya TM Siiteri PK Vigne JL Licht P Pavgi S Frank LG. A mechanism for virilization of female spotted hyenas in utero. Science (1993) 260(5116):1929, 31. doi: 10.1126/science.8391165 8391165

[38] Drea CM. Endocrine correlates of pregnancy in the ring-tailed lemur (*Lemur catta*): Implications for the masculinization of daughters. Hormones Behavior. (2011) 59:417–27. doi: 10.1016/j.yhbeh.2010.09.011 20932838

[39] Brown GR Spencer KA. Steroid hormones, stress and the adolescent brain: a comparative perspective. Neuroscience. (2013) :249:115–28. doi: 10.1016/j.neuroscience.2012.12.016 23262238

[40] Staub NL De Beer M. The role of androgens in female vertebrates. Gen Comp Endocrinol. (1997) 108:1–24. doi: 10.1006/gcen.1997.6962 9378263

[41] Dantzer B Swanson EM. Mediation of vertebrate life histories via insulin-like growth factor-1. Biol Rev. (2012) 87:414–29. doi: 10.1111/j.1469-185X.2011.00204.x 21981025

[42] Bartke A Sun LY Longo V. Somatotropic signaling: trade-offs between growth, reproductive development, and longevity. Physiol Rev. (2013) 93:571–98. doi: 10.1152/physrev.00006.2012 PMC3768106 23589828

[43] Al-Samerria S Radovick S. The role of insulin-like growth factor-1 (IGF-1) in the control of neuroendocrine regulation of growth. Cells. (2021) 10:2664. doi: 10.3390/cells10102664 34685644 PMC8534318

[44] Veldhuis JD Frystyk J Iranmanesh A Ørskov H. Testosterone and estradiol regulate free insulin-like growth factor I (IGF-I), IGF binding protein 1 (IGFBP-1), and dimeric IGF-I/IGFBP-1 concentrations. J Clin Endocrinol Metab. (2005) 90:2941–7. doi: 10.1210/jc.2004-1314 PMC1289262 15713723

[45] Bernstein RM Setchell JM Verrier D Knapp LA. Maternal effects and the endocrine regulation of mandrill growth. Am J Primatol. (2012) 74:890–900. doi: 10.1002/ajp.22038 22696170

[46] Vitale G Pellegrino G Vollery M Hofland LJ. Role of IGF-1 system in the modulation of longevity: controversies and new insights from a centenarians’ perspective. Front Endocrinol. (2019) 10:27. doi: 10.3389/fendo.2019.00027 PMC6367275 30774624

[47] Ketterson ED Nolan V. Hormones and life histories: an integrative approach. Am Naturalist. (1992) 140:S33–62. doi: 10.1086/285396 19426026

[48] Hau M. Regulation of male traits by testosterone: implications for the evolution of vertebrate life histories. BioEssays. (2007) 29:133–44. doi: 10.1002/bies.20524 17226801

[49] Kutsukake N Clutton-Brock TH. Aggression and submission reflect reproductive conflict between females in cooperatively breeding meerkats *Suricata suricatta* . Behav Ecol Sociobiol. (2006) 59:541–8. doi: 10.1007/s00265-005-0079-7

[50] Gómez JM Verdú M González-Megías A Méndez M. The phylogenetic roots of human lethal violence. Nature. (2016) 538:233–7. doi: 10.1038/nature19758 27680701

[51] Clutton-Brock TH Hodge SJ Spong G Russell AF Jordan NR Bennett NC. Intrasexual competition and sexual selection in cooperative mammals. Nature. (2006) 444:1065–8. doi: 10.1038/nature05386 17183322

[52] Davies CS Smyth KN Greene LK Walsh DA Mitchell J Clutton-Brock T. Exceptional endocrine profiles characterise the meerkat: sex, status, and reproductive patterns. Sci Rep. (2016) 6:35492. doi: 10.1038/srep35492 27752129 PMC5067592

[53] Drea CM Davies CS. Meerkat manners: Endocrine mediation of female dominance and reproductive control in a cooperative breeder. Hormones Behavior. (2022) :145:105245. doi: 10.1016/j.yhbeh.2022.105245 35988450

[54] Dimac-Stohl KA Davies CS Grebe NM Stonehill AC Greene LK Mitchell J. Incidence and biomarkers of pregnancy, spontaneous abortion, and neonatal loss during an environmental stressor: implications for female reproductive suppression in the cooperatively breeding meerkat. Physiol Behavior. (2018) :193:90–100. doi: 10.1016/j.physbeh.2017.11.011 29730033

[55] Doolan SP Macdonald DW. Band structure and failures of reproductive suppression in a cooperatively breeding carnivore, the slender-tailed meerkat (*Suricata suricatta*). Behaviour. (1997) 134:827–48. https://www.jstor.org/stable/4535474.

[56] Smyth KN Greene LK Clutton-Brock T Drea CM. Androgens predict parasitism in female meerkats: a new perspective on a classic trade-off. Biol Letters. (2016) 12:20160660. doi: 10.1098/rsbl.2016.0660 PMC5095201 28120802

[57] Smyth KN Caruso NM Davies CS Clutton-Brock TH Drea CM. Social and endocrine correlates of immune function in meerkats: implications for the immunocompetence handicap hypothesis. R Soc Open Sci. (2018) 5:180435. doi: 10.1098/rsos.180435 30225031 PMC6124081

[58] MacLeod KJ Clutton-Brock TH. No evidence for adaptive sex ratio variation in the cooperatively breeding meerkat, Suricata suricatta. Anim Behav. (2013) 85:645–53. doi: 10.1016/j.anbehav.2012.12.028

[59] Holekamp KE Strauss ED. Aggression and dominance: an interdisciplinary overview. Curr Opin Behav Sci. (2016) 12:44–51. doi: 10.1016/j.cobeha.2016.08.005

[60] Lewin N Swanson EM Williams BL Holekamp KE. Juvenile concentrations of IGF-1 predict life-history trade-offs in a wild mammal. Funct Ecol. (2017) 31:894–902. doi: 10.1111/1365-2435.12808

[61] Russell AF Clutton-Brock TH Brotherton PN Sharpe LL McIlrath G Dalerum FD. Factors affecting pup growth and survival in co-operatively breeding meerkats *Suricata suricatta* . J Anim Ecol. (2002) 71(4): 700–9. doi: 10.1046/j.1365-2656.2002.00636.x

[62] Schwabl H. Maternal testosterone in the avian egg enhances postnatal growth. Comp Biochem Physiol Part A: Physiol. (1996) 114:271–6. doi: 10.1016/0300-9629(96)00009-6 8759148

[63] Smyth KN Caruso NM Stonehill AC Clutton-Brock TH Drea CM. Maternal androgens in dominant meerkats (*Suricata suricatta*) reduce juvenile offspring health and survivorship. Authorea. doi: 10.22541/au.172561356.63212791/v1 PMC11581709 39575152

[64] Doolan SP MacDonald DW. Diet and foraging behaviour of group-living meerkats, *Suricata suricatta*, in the Southern Kalahari. J Zool. (1996) 239:697–716. doi: 10.1111/j.1469-7998.1996.tb05472.x

[65] delBarco-Trillo J Greene LK Goncalves IB Fenkes M Wisse JH Drewe JA. Beyond aggression: Androgen-receptor blockade modulates social interaction in wild meerkats. Hormones Behavior. (2016) 78:95–106. doi: 10.1016/j.yhbeh.2015.11.001 26545817

[66] Griffin AS Pemberton JM Brotherton PN McIlrath G Gaynor D Kansky R. A genetic analysis of breeding success in the cooperative meerkat (*Suricata suricatta*). Behav Ecol. (2003) 14:472–80. doi: 10.1093/beheco/arg040

[67] Clutton-Brock TH Gaynor D Kansky R MacColl AD McIlrath G Chadwick P. Costs of cooperative behaviour in suricates (*Suricata suricatta*). Proc R Soc London Ser B: Biol Sci. (1998) 265:185–90. doi: 10.1098/rspb.1998.0281 PMC1688874 9493405

[68] English S Huchard E Nielsen JF Clutton-Brock TH. Early growth, dominance acquisition and lifetime reproductive success in male and female cooperative meerkats. Ecol Evolution. (2013) 3:4401–7. doi: 10.1002/ece3.820 PMC3856740 24340181

[69] Clutton-Brock TH Hodge SJ Flower TP Spong GF Young AJ. Adaptive suppression of subordinate reproduction in cooperative mammals. Am Naturalist. (2010) 176:664–73. doi: 10.1086/656492 20846043

[70] Mares R Bateman AW English S Clutton-Brock TH Young AJ. Timing of predispersal prospecting is influenced by environmental, social and state-dependent factors in meerkats. Anim Behav. (2014) 88:185–93. doi: 10.1016/j.anbehav.2013.11.025

[71] Santema P Teitel Z Manser M Bennett N Clutton-Brock T. Effects of cortisol administration on cooperative behavior in meerkat helpers. Behav Ecol. (2013) 24:1122–7. doi: 10.1093/beheco/art039

[72] Wood SN. Generalized Additive Models: An Introduction with R. 2nd ed. New York: Chapman and Hall/CRC (2017). doi: 10.1201/9781315370279

[73] R Core Team. R: A Language and Environment for Statistical Computing. Vienna, Austria, New York: R Foundation for Statistical Computing (2023). Available at: https://www.R-project.org/.

[74] Russell AF Carlson AA McIlrath GM Jordan NR Clutton-Brock T. Adaptive size modification by dominant female meerkats. Evolution. (2004) 58:1600–7. doi: 10.1111/j.0014-3820.2004.tb01739.x (Accessed: April 15, 2024). 15341161

[75] Burnham KP Anderson DR. Model selection and multimodel inference: a practical information-theoretic approach. 2nd ed. New York, USA: Springer (2002). doi: 10.1007/978-0-387-22456-5_6

[76] Wood SN. Fast stable restricted maximum likelihood and marginal likelihood estimation of semiparametric generalized linear models. J R Stat Society: Ser B (Statistical Methodology). (2011) 73:3–36. doi: 10.1111/j.1467-9868.2010.00749

[77] Hartig F. DHARMa: residual diagnostics for hierarchical (multi-level/mixed) regression models. R package version 024 (2017). Available online at: http://florianhartig.github.io/DHARMa/ (Accessed: April 15, 2024).

[78] Wickham H. ggplot2: Elegant Graphics for Data Analysis. New York: Springer-Verlag (2016). Available at: https://ggplot2.tidyverse.org (Accessed: April 15, 2024).

[79] Brooks ME Kristensen K van Benthem KJ Magnusson A Berg CW Nielsen A. glmmTMB balances speed and flexibility among packages for zero-inflated generalized linear mixed modeling. R J. (2017) 9:378–400. doi: 10.3929/ethz-b-000240890

[80] Adkins-Regan E Abdelnabi M Mobarak M Ottinger MA. Sex steroid levels in developing and adult male and female zebra finches (*Poephila guttata*). Gen Comp Endocrinol. (1990) 78:93–109. doi: 10.1016/0016-6480(90)90051-M 2332151

[81] Dhakal P Hirama A Nambo Y Harada T Sato F Nagaoka K. Circulating pituitary and gonadal hormones in spring-born Thoroughbred fillies and colts from birth to puberty. J Reprod Dev. (2012) 58:522–30. doi: 10.1262/jrd.2011-025 22673032

[82] Schulz KM Sisk CL. The organizing actions of adolescent gonadal steroid hormones on brain and behavioral development. Neurosci Biobehav Rev. (2016) 70:148–58. doi: 10.1016/j.neubiorev.2016.07.036 PMC5074860 27497718

[83] Power ML Schulkin J. Maternal regulation of offspring development in mammals is an ancient adaptation tied to lactation. Appl Trans Genomics. (2013) 2:55–63. doi: 10.1016/j.atg.2013.06.001 PMC5121250 27896056

[84] Grosvenor CE Picciano MF Baumrucker CR. Hormones and growth factors in milk. Endocrine Rev. (1993) 14:710–28. doi: 10.1210/edrv-14-6-710 8119234

[85] Gaiani R Chiesa F Mattioli M Nannetti G Galeati G. Androstenedione and testosterone concentrations in plasma and milk of the cow throughout pregnancy. Reproduction. (1984) 70:559. doi: 10.1530/jrf.0.0700055 6694152

[86] Licht P Frank LG Pavgi S Yalcinkaya TM Siiteri PK Glickman SE. Hormonal correlates of ‘masculinization’ in female spotted hyaenas (*Crocuta crocuta*). 2. Maternal and fetal steroids. Reproduction. (1992) 95:463–74. doi: 10.1530/jrf.0.0950463 1518002

[87] Grebe NM Sheikh A Drea CM. Integrating the female masculinization and challenge hypotheses: Female dominance, male deference, and seasonal hormone fluctuations in adult blue-eyed black lemurs (*Eulemur flavifrons*). Hormones Behavior. (2022) 139:105108. doi: 10.1016/j.yhbeh.2022.105108 35033896

[88] Edwards PD Arguelles DA MastroMonaco GF Holmes MM. Queen pregnancy increases group estradiol levels in cooperatively breeding naked mole-rats. Integr Comp Biol. (2021) 61:1841–51. doi: 10.1093/icb/icab106 34048558

[89] Wynne-Edwards KE. Hormonal changes in mammalian fathers. Hormones Behavior. (2001) 40:139–45. doi: 10.1006/hbeh.2001.1699 11534974

[90] Numan M Insel TR. Paternal behavior. Neurobiol Parental Behav. (2003), 246–67. (New York: Springer-Verlag).

[91] Carlson AA Manser MB Young AJ Russell AF Jordan NR McNeilly AS. Cortisol levels are positively associated with pup-feeding rates in male meerkats. Proc R Soc B: Biol Sci. (2006) 273:571–7. doi: 10.1098/rspb.2005.3087 PMC1560054 16537128

[92] Wingfield JC Hegner RE Dufty AM Jr. Ball GF. The “challenge hypothesis”: theoretical implications for patterns of testosterone secretion, mating systems, and breeding strategies. Am Naturalist. (1990) 136:829–46. doi: 10.1086/285134

[93] Medger K Bennett NC Ganswindt SB Ganswindt A Hart DW. Changes in prolactin, cortisol and testosterone concentrations during queen succession in a colony of naked mole-rats (*Heterocephalus glab*er): a case study. Sci Nature. (2019) 106:1–7. doi: 10.1007/s00114-019-1621-1 31089819

[94] Clutton-Brock TH Hodge SJ Flower TP. Group size and the suppression of subordinate reproduction in Kalahari meerkats. Anim Behav. (2008) 76:689–700. doi: 10.1016/j.anbehav.2008.03.015

[95] Paniw M Maag N Cozzi G Clutton-Brock T Ozgul A. Life history responses of meerkats to seasonal changes in extreme environments. Science. (2019) 363:631–5. doi: 10.1126/science.aau5905 30733418

[96] Rotics S Clutton-Brock T. Group size increases inequality in cooperative behaviour. Proc R Soc B. (2021) 288:20202104. doi: 10.1098/rspb.2020.2104 PMC7934907 33593194

[97] Dunshea FR King RH Campbell RG Sainz RD Kim YS. Interrelationships between sex and ractopamine on protein and lipid deposition in rapidly growing pigs. J Anim Sci. (1993) 71:2919–30. doi: 10.2527/1993.71112919x 7903662

[98] Ueberschlag-Pitiot V Stantzou A Messéant J Lemaitre M Owens DJ Noirez P. Gonad-related factors promote muscle performance gain during postnatal development in male and female mice. Am J Physiol-Endocrinol Metab. (2017) 313:E12–25. doi: 10.1152/ajpendo.00446.2016 28351832

[99] Lewin NS. Mechanisms mediating life history traits in the spotted hyena (*Crocuta crocuta*). Doctoral Dissertation. Michigan State University (2017).

[100] Engström E Niklasson A Wikland K Ewald U Hellström A. The role of maternal factors, postnatal nutrition, weight gain, and gender in regulation of serum IGF-I among preterm infants. Pediatr Res. (2005) 57:605–10. doi: 10.1203/01.PDR.0000155950.67503.BC 15695599

[101] Crespi EJ Steckler TL MohanKumar PS Padmanabhan V. Prenatal exposure to excess testosterone modifies the developmental trajectory of the insulin-like growth factor system in female sheep. J Physiol. (2006) 572:119–30. doi: 10.1113/jphysiol.2005.103929 PMC1779643 16484301

[102] DeHaan KC Berger LL Bechtel PJ Kesler DJ McKeith FK Thomas DL. Effect of prenatal testosterone treatment on nitrogen utilization and endocrine status of ewe lambs. J Anim Sci. (1990) 68:4100–8. doi: 10.2527/1990.68124100x 2286551

[103] Aldrich SL Berger LL Kesler DJ Nash TG McCusker RH. Effects of prenatal androgenization and postnatal steroid treatment on growth hormone, insulin-like growth factor I and II, insulin, thyroxine, and triidothyronine concentrations in beef heifers. J Anim Sci. (1996) 74:420–8. doi: 10.2527/1996.742420x 8690679

[104] Greer KA Hughes LM Masternak MM. Connecting serum IGF-1, body size, and age in the domestic dog. AGE. (2011) 33:475–83. doi: 10.1007/s11357-010-9182-4 PMC3168604 20865338

[105] Ong K Kratzsch J Kiess W Dunger D. Circulating IGF-I levels in childhood are related to both current body composition and early postnatal growth rate. J Clin Endocrinol Metab. (2002) 87:1041–4. doi: 10.1210/jcem.87.3.8342 11889159

[106] Yakar S Wu Y Setser J Rosen CJ. The role of circulating IGF-I. Endocrine. (2002) 19:239–48. doi: 10.1385/ENDO:19:3:239 12624423

[107] Fliesen T Maiter D Gerard G Underwood LE Maes M Ketelslegers JM. Reduction of serum insulin-like growth factor-I by dietary protein restriction is age dependent. Pediatr Res. (1989) 26:415–9. doi: 10.1203/00006450-198911000-00010 2812891

[108] Clutton-Brock TH Russell AF Sharpe LL Brotherton PN McIlrath GM White S. Effects of helpers on juvenile development and survival in meerkats. Science. (2001) 293:2446–9. doi: 10.1126/science.1061274 11577235

[109] Smink J Gresnigt M Hamers N Koedam J Berger R Van Buul-Offers S. Short-term glucocorticoid treatment of prepubertal mice decreases growth and IGF-I expression in the growth plate. J Endocrinol. (2003) 177:381–8. doi: 10.1677/joe.0.1770381 12773118

[110] Lodjak J Tilgar V Mägi M. Does the interaction between glucocorticoids and insulin-like growth factor 1 predict nestling fitness in a wild passerine? Gen Comp Endocrinol. (2016) 225:149–54. doi: 10.1016/j.ygcen.2015.10.016 26519758

[111] Lodjak J Verhulst S. Insulin-like growth factor 1 of wild vertebrates in a life-history context. Mol Cell Endocrinol. (2020) 518:110978. doi: 10.1016/j.mce.2020.11097 32798584

[112] Uller T Astheimer L Olsson M. Consequences of maternal yolk testosterone for offspring development and survival: experimental test in a lizard. Funct Ecol. (2007) 1:544–51. doi: 10.1111/j.1365-2435.2007.01264.x

[113] Cram DL Monaghan P Gillespie R Clutton-Brock T. Effects of early-life competition and maternal nutrition on telomere lengths in wild meerkats. Proc R Soc B: Biol Sci. (2017) 284:20171383. doi: 10.1098/rspb.2017.1383 PMC5577495 28855370

[114] Uller T Olsson M. Prenatal exposure to testosterone increases ectoparasite susceptibility in the common lizard (*Lacerta vivipara*). Proc R Soc London Ser B: Biol Sci. (2003) 270:1867–70. doi: 10.1098/rspb.2003.2451 PMC1691442 12964990

[115] Andersson S Uller T Lõhmus M Sundström F. Effects of egg yolk testosterone on growth and immunity in a precocial bird. J Evolutionary Biol. (2004) 17:501–5. doi: 10.1111/j.1420-9101.2004.00706.x 15149393

[116] Groothuis TG Müller W von Engelhardt N Carere C Eising C. Maternal hormones as a tool to adjust offspring phenotype in avian species. Neurosci Biobehav Rev. (2005) 29:329–52. doi: 10.1016/j.neubiorev.2004.12.002 15811503

[117] Pavitt AT Walling CA McNeilly AS Pemberton JM Kruuk LE. Variation in early-life testosterone within a wild population of red deer. Funct Ecol. (2014) 28:1224–34. doi: 10.1111/1365-2435.12260

[118] Patterson S Drewe JA Pfeiffer DU Clutton-Brock TH. Social and environmental factors affect tuberculosis related mortality in wild meerkats. J Anim Ecol. (2017) 86:442–50. doi: 10.1111/1365-2656.12649 PMC5413830 28186336

[119] Van de Ven TM Fuller A Clutton-Brock TH. Effects of climate change on pup growth and survival in a cooperative mammal, the meerkat. Funct Ecol. (2020) 34:194–202. doi: 10.1111/1365-2435.13468

[120] Groenewoud F Clutton-Brock T. Meerkat helpers buffer the detrimental effects of adverse environmental conditions on fecundity, growth and survival. J Anim Ecol. (2021) 90:641–52. doi: 10.1111/1365-2656.13396 33241582

[121] Lutermann H Young AJ Bennett NC. Reproductive status and testosterone among females in cooperative mole-rat societies. Gen Comp Endocrinol. (2013) 187:60–5. doi: 10.1016/j.ygcen.2013.03.026 23583770

[122] French JA Mustoe AC Cavanaugh J Birnie AK. The influence of androgenic steroid hormones on female aggression in ‘atypical’ mammals. Philos Trans R Soc B: Biol Sci. (2013) 368:20130084. doi: 10.1098/rstb.2013.0084 PMC3826213 24167314

[123] Drea CM Grebe NM. Intraspecific aggression and social dominance. In: The Routledge International Handbook of Comparative Psychology. Routledge (2022). p. 160–74.

[124] Sun M Maliqueo M Benrick A Johansson J Shao R Hou L. Maternal androgen excess reduces placental and fetal weights, increases placental steroidogenesis, and leads to long-term health effects in their female offspring. Am J Physiol-Endocrinol Metab. (2012) 303:E1373–85. doi: 10.1152/ajpendo.00421.2012 23047983

[125] Browne P Place NJ Vidal JD Moore IT Cunha GR Glickman SE. Endocrine differentiation of fetal ovaries and testes of the spotted hyena (*Crocuta crocuta*): timing of androgen-independent versus androgen-driven genital development. Reproduction. (2006) 132:649–59. doi: 10.1530/rep.1.0112 17008476

[126] Rosvall KA Bergeon Burns CM Barske J Goodson JL Schlinger BA Sengelaub DR. Neural sensitivity to sex steroids predicts individual differences in aggression: implications for behavioural evolution. Proc R Soc B: Biol Sci. (2012) 279:3547–55. doi: 10.1098/rspb.2012.0442 PMC3396890 22673360

[127] Conte FA Grumbach MM Ito Y Fisher CR Simpson ER. A syndrome of female pseudohermaphrodism, hypergonadotropic hypogonadism, and multicystic ovaries associated with missense mutations in the gene encoding aromatase (P450arom). J Clin Endocrinol Metab. (1994) 78:1287–92. doi: 10.1210/jcem.78.6.8200927 8200927

[128] Gomes LG Bachega TA Mendonca BB. Classic congenital adrenal hyperplasia and its impact on reproduction. Fertility Sterility. (2019) 111:7–12. doi: 10.1016/j.fertnstert.2018.11.037 30611420

[129] Filippou P Homburg R. Is foetal hyperexposure to androgens a cause of PCOS? Hum Reprod. (2017) 23:421–32. doi: 10.1093/humupd/dmx013 28531286

