# Supplementary material for: Androgen-mediated maternal effects and trade-offs: postnatal hormone development, growth, and survivorship in wild meerkats
Source: Front Endocrinol (Lausanne). 2024 Sep 30;15:1418056. doi: 10.3389/fendo.2024.1418056 (PMC11471613; doi:10.3389/fendo.2024.1418056)
Supplement: Supplementary file 1 [file DataSheet1.docx]

**Androgen-mediated maternal effects and trade-offs: postnatal hormone development, growth, and survivorship in wild meerkats**

Charli S. Davies^1,2,†,‡^, Caroline L. Shearer^1,3,†^, Lydia K. Greene ^1,2,3,§^, Jessica Mitchell^1,2, ||^, Debbie Walsh^1,2^, Vivian C. Goerlich^1,2,#^, Tim H. Clutton-Brock^2,4,5^, and Christine M. Drea^1,2,3,6,*^

^1^ Department of Evolutionary Anthropology, Duke University, Durham, NC, United States.

^2^ Kalahari Research Trust, Kuruman River Reserve, Northern Cape, South Africa.

^3^ University Program in Ecology, Duke University, Durham, NC, United States.

^4^ Department of Zoology, University of Cambridge, Cambridge, United Kingdom.

^5^ Mammal Research Institute, University of Pretoria, 0002 Pretoria, South Africa.

^6^ Department of Biology, Duke University, Durham, NC, United States.

† These authors share first authorship

*Correspondence: Christine Drea, [cdrea@duke.edu](mailto:cdrea@duke.edu)

Present addresses:

‡ Department of Biological and Environmental Science, University of Jyväskylä, Jyväskylä, Finland.

§ Department of Biology, Duke University, Durham, NC, United States

|| Global Academy for Agriculture and Food Science, Veterinary School, University of Edinburgh, UK.

# Animals in Science and Society, Department of Population Health Sciences, Faculty of Veterinary Medicine, Utrecht University, Utrecht, the Netherlands

**Abstract**

**Introduction:** Mammalian reproductive and somatic development is regulated by steroid hormones, growth hormone (GH), and insulin-like growth factor-1 (IGF-1). Based largely on information from humans, model organisms, and domesticated animals, testosterone (T) and the GH/IGF-1 system activate sexually differentiated development, promoting male-biased growth, often at a cost to health and survivorship. To test if augmented prenatal androgen exposure in females produces similar developmental patterns and trade-offs, we examine maternal effects in wild meerkats (*Suricata suricatta*), a non-model species in which adult females naturally, albeit differentially by status, express exceptionally high androgen concentrations, particularly during pregnancy. In this cooperative breeder, the early growth of daughters predicts future breeding status and reproductive success.

**Methods:** We examine effects of normative and experimentally induced variation in maternal androgens on the ontogenetic patterns in offspring reproductive hormones (androstenedione, A4; T; estradiol, E2), IGF-1, growth from pup emergence at 1 month to puberty at 1 year, and survivorship. Specifically, we compare the male and female offspring of dominant control (DC or high-T), subordinate control (SC or lower-T), and dominant treated (DT or blocked-T) dams, the latter having experienced antiandrogen treatment in late gestation.

**Results:** Meerkat offspring showed sex differences in absolute T and IGF-1 concentrations, developmental rates of A4 and E2 expression, and survivorship — effects that were sometimes socially or environmentally modulated. Atypical for mammals were the early male bias in T that disappeared by puberty, the absence of sex differences in A4 and E2, and the female bias in IGF-1. Food availability was linked to steroid concentrations in females and to IGF-1, potentially growth, and survival in both sexes. Maternal treatment significantly affected rates of T, E2, and IGF-1 expression, and weight, with marginal effects on survivorship; offspring of DT dams showed peak IGF-1 concentrations and the best survivorship.

**Discussion:** Maternal effects thus impact offspring development in meerkats, with associated trade-offs: Whereas prenatal androgens modify postnatal reproductive and somatic physiology, benefits associated with enhanced competitiveness in DC lineages may have initial costs of reduced IGF-1, delay in weight gain, and decreased survivorship. These novel data further confirm the different evolutionary and mechanistic pathways to cooperative breeding and call for greater consideration of natural endocrine variation in both sexes.

**Keywords:**female masculinization, flutamide, IGF-1, life-history trade-offs, ontogeny, prenatal programming, sex steroids, sexual differentiation

**Table S1 |** Predictors of serum hormone concentrations in meerkat (*Suricata suricatta*) offspring, based on top models. The offspring were from one of three maternal treatment groups: Dominant Control (DC) had naturally greater androgen exposure in utero; Subordinate Control (SC) had naturally lower androgen exposure; Dominant Treated (DT) had experimentally blocked androgen receptors during late gestation.

| **Hormone** | **Model information** | | | |
| --- | --- | --- | --- | --- |
| Androstenedione, A_4_^*^  *n* = 249 | Parametric terms | Estimate (SE) | *t* | *P* |
|  | Sex: Female | 0.17 (0.18) | 0.92 | 0.358 |
|  | Maternal treatment: SC | - 0.21 (0.22) | -1.24 | 0.325 |
|  | Maternal treatment: DT | -0.12 (0.27) | -0.44 | 0.661 |
|  | Smooth terms | EDF | *F* | *P* |
|  | **Age (Male)** | **3.421** | **3.34** | **0.017** |
|  | **Age (Female)** | **2.615** | **3.36** | **0.019** |
|  | Total monthly rainfall | 1.704 | 1.31 | 0.354 |
|  | **Body condition residuals** | **5.582** | **6.87** | **< 0.001** |
| Testosterone, T^*^  *n* = 236 | Parametric terms | Estimate (SE) | *t* | *P* |
|  | **Sex: Female** | **-1.60 (0.24)** | **-6.78** | **< 0.001** |
|  | Maternal treatment: SC | -0.18 (0.32) | -0.57 | 0.567 |
|  | Maternal treatment: DT | 0.03 (0.41) | 0.08 | 0.934 |
|  | Smooth terms | EDF | *F* | *P* |
|  | Age (Male) | 1.957 | 2.34 | 0.256 |
|  | *Age (Female)* | *2.25* | *2.24* | *0.094* |
|  | Age (DC) | 1.002 | 1.78 | 0.184 |
|  | Age (SC) | 1.000 | 1.16 | 0.283 |
|  | **Age (DT)** | **1.557** | **3.50** | **0.032** |
|  | Total monthly rainfall (Male) | 1.352 | 1.12 | 0.421 |
|  | **Total monthly rainfall (Female)** | **1.745** | **3.84** | **0.031** |
|  | **Body condition residuals** | **2.692** | **28.04** | **< 0.001** |
| Estradiol, E_2_  *n* = 160 | Parametric terms | Estimate (SE) | *t* | *P* |
|  | Sex: Female | 0.20 (0.17) | 1.15 | 0.252 |
|  | Maternal treatment: SC | -0.21 (0.29) | -0.71 | 0.477 |
|  | Maternal treatment: DT | 0.06 (0.38) | 0.15 | 0.881 |
|  | Total monthly rainfall | 0.001 (0.003) | 0.40 | 0.688 |
|  | **Clan size** | **-0.04 (0.01)** | **-3.15** | **0.002** |
|  | **Total monthly rainfall*Sex** | **-0.01 (0.005)** | **-3.03** | **0.003** |
|  | Smooth terms | EDF | *F* | *P* |
|  | Age (Male) | 1.000 | 0.88 | 0.351 |
|  | **Age (Female)** | **2.501** | **8.99** | **0.015** |
|  | Age (DC) | 0.073 | 0.002 | 0.999 |
|  | *Age (SC)* | *1.000* | *3.47* | *0.065* |
|  | **Age (DT)** | **1.000** | **7.10** | **0.009** |
|  | **Body condition residuals** | **1.002** | **3.97** | **0.048** |
| Insulin-like growth factor-1, IGF-1  *n* = 155 | Parametric terms | Estimate (SE) | *t* | *P* |
|  | **Sex: Female** | **0.26 (0.11)** | **2.31** | **0.023** |
|  | Maternal treatment: SC | 0.15 (0.17) | 0.87 | 0.387 |
|  | Maternal treatment: DT | 0.26 (0.22) | 1.18 | 0.242 |
|  | **Total monthly rainfall** | **0.01 (0.002)** | **4.75** | **< 0.001** |
|  | **Body condition residuals** | **0.003 (0.001)** | **2.94** | **0.004** |
|  | **Clan size** | **-0.03 (0.01)** | **-2.81** | **0.006** |
|  | **Total monthly rainfall*Sex** | **-0.01 (0.003)** | **-2.07** | **0.041** |
|  | Smooth terms | EDF | *F* | *P* |
|  | **Age (DC)** | **2.462** | **3.30** | **0.040** |
|  | **Age (SC)** | **3.131** | **3.04** | **0.016** |
|  | *Age (DT)* | *2.437* | *2.47* | *0.069* |

Note. These results derive from generalized additive models (GAM). Random effects include individual and litter. All comparisons were made against the indicated levels of each factor: Sex = male; Maternal treatment = dominant control. Terms in parentheses are levels of the “by” factor for a given smooth. Estimates are on log scale. The empirical distribution function (EDF) is related to the non-linearity of the smooth term, with values close to 1 approaching linear.  *t* = Wald parametric test statistic. *F* = test statistic for confidence interval of smooth terms. Bolding indicates significant terms (*P* < 0.05); italics indicate trends (*P* < 0.10).

^*^Clan size was excluded from the model because it did not reach significance or improve the deviance explained by at least 1%.

**Table S2 |** Predictors of serum hormone concentrations in meerkat (*Suricata suricatta*) offspring at 12 months. The offspring were from one of three maternal treatment groups: Dominant Control (DC) had naturally greater androgen exposure in utero; Subordinate Control (SC) had naturally lower androgen exposure; Dominant Treated (DT) had experimentally blocked androgen receptors during late gestation.

| **Hormone** | **Model information** | | | |
| --- | --- | --- | --- | --- |
| Androstenedione, A_4_  *n* = 43 | Parametric terms | Estimate (SE) | *z* | *P* |
|  | *Sex: Female* | *0.83 (0.45)* | *1.85* | *0.064* |
|  | Maternal treatment: SC | -0.24 (0.48) | -0.50 | 0.619 |
|  | Maternal treatment: DT | -0.25 (0.57) | -0.45 | 0.656 |
|  | *Total monthly rainfall* | *0.07 (0.04)* | *1.90* | *0.057* |
|  | Body condition residuals | 0.003 (0.003) | 1.20 | 0.229 |
| Testosterone, T  *n* = 33 | Parametric terms | Estimate (SE) | *z* | *P* |
|  | Sex: Female | -0.67 (1.11) | -0.61 | 0.542 |
|  | Maternal treatment: SC | 0.17 (0.66) | 0.25 | 0.801 |
|  | Maternal treatment: DT | 0.64 (0.76) | 0.84 | 0.403 |
|  | Total monthly rainfall | 0.06 (0.04) | 1.46 | 0.144 |
|  | Total monthly rainfall*Sex | 0.005 (0.005) | 1.06 | 0.288 |
|  | Body condition residuals | 0.15 (0.24) | 0.65 | 0.518 |
| Estradiol, E_2_  *n* = 36 | Parametric terms | Estimate (SE) | *z* | *P* |
|  | Sex: Female | 0.69 (0.47) | 1.46 | 0.145 |
|  | *Maternal treatment: SC* | *-0.64 (0.36)* | *-1.80* | *0.072* |
|  | Maternal treatment: DT | 0.14 (0.38) | 0.38 | 0.706 |
|  | **Total monthly rainfall** | **0.08 (0.02)** | **3.43** | **<0.001** |
|  | **Clan size** | **-0.10 (0.03)** | **-3.37** | **<0.001** |
|  | Total monthly rainfall*Sex | -0.05 (0.08) | -0.63 | 0.526 |
|  | **Body condition residuals** | **0.004 (0.002)** | **2.15** | **0.032** |
| Insulin-like growth factor 1, IGF-1  *n* = 33 | Parametric terms | Estimate (SE) | *t* | *P* |
|  | Sex: Female | 0.38 (0.24) | 1.58 | 0.114 |
|  | Maternal treatment: SC | 0.10 (0.15) | -0.64 | 0.523 |
|  | Maternal treatment: DT | 0.24 (0.16) | 1.54 | 0.124 |
|  | **Total monthly rainfall** | **0.04 (0.01)** | **3.83** | **<0.001** |
|  | **Body condition residuals** | **0.005 (0.001)** | **5.80** | **<0.001** |
|  | **Clan size** | **-0.03 (0.01)** | **-2.10** | **0.036** |
|  | Total monthly rainfall*Sex | -0.02 (0.05) | -0.35 | 0.723 |

Note. These results derive from generalized linear mixed models (GLMMs). Random effects include individual. All comparisons were made against the indicated levels of each factor: Sex = male; Maternal treatment = dominant control. Estimates are on log scale. *z* = test statistic. Bolding indicates significant terms (*P* < 0.05); italics indicate trends (*P* < 0.10). Model terms were based on top GAM model for each hormone in Table S1.

**Table S3 |** Predictors of weight in meerkat (*Suricata suricatta*) offspring. The offspring were from one of three maternal treatment groups: Dominant Control (DC) had naturally greater androgen exposure in utero; Subordinate Control (SC) had naturally lower androgen exposure; Dominant Treated (DT) had experimentally blocked androgen receptors during late gestation.

| Parametric terms | Estimate (SE) | *t* | *P* |
| --- | --- | --- | --- |
| Sex: Female | 3.62 (4.41) | 0.82 | 0.412 |
| Maternal treatment: SC | 3.99 (21.03) | 0.19 | 0.850 |
| Maternal treatment: DT | -0.59 (28.86) | -0.02 | 0.984 |
| *Total monthly rainfall* | *-0.15 (0.09)* | *-1.68* | *0.094* |
| **Average hindfoot length** | **5.81 (0.66)** | **8.76** | **< 0.001** |
| Smooth terms | EDF | *F* | *P* |
| **Age (DC)** | **3.466** | **44.33** | **< 0.001** |
| **Age (SC)** | **3.583** | **67.67** | **< 0.001** |
| **Age (DT)** | **2.540** | **35.26** | **< 0.001** |
| **Clan size** | **1.926** | **18.28** | **< 0.001** |

Note. These results derive from a generalized additive model (GAM). Random effects include individual (*n* = 299) and litter. All comparisons were made against the indicated levels of each factor: Sex = male; Maternal treatment = dominant control. Terms in parentheses are levels of the “by” factor for a given smooth. Estimates are on response scale. The empirical distribution function (EDF) is related to the non-linearity of the smooth term, with values close to 1 approaching linear. *t* = Wald parametric test statistic. *F* = test statistic for confidence interval of smooth terms. Bolding indicates significant terms (*P* < 0.05); italics indicate trends (*P* < 0.10).

**Table S4 |** Predictors of weight at 12 months in meerkat (*Suricata suricatta*) offspring. The offspring were from one of three maternal treatment groups: Dominant Control (DC) had naturally greater androgen exposure in utero; Subordinate Control (SC) had naturally lower androgen exposure; Dominant Treated (DT) had experimentally blocked androgen receptors during late gestation.

| Parametric terms | Estimate (SE) | *t* | *P* |
| --- | --- | --- | --- |
| Sex: Female | -7.46 (23.19) | -0.32 | 0.748 |
| Maternal treatment: SC | -21.17 (26.19) | -0.81 | 0.419 |
| Maternal treatment: DT | -31.55 (33.52) | -0.94 | 0.347 |
| Total monthly rainfall | 1.59 (2.02) | 0.79 | 0.430 |
| **Average hindfoot length** | **15.92 (5.92)** | 2.69 | **0.007** |
| **Clan size** | -1.06 (2.15) | -0.49 | 0.623 |

Note. These results derive from generalized linear mixed models (GLMMs). Random effects include individual (*n* = 39). All comparisons were made against the indicated levels of each factor: Sex = male; Maternal treatment = dominant control. Estimates are on log scale. *z* = test statistic. Bolding indicates significant terms (*P* < 0.05); italics indicate trends (*P* < 0.10). Model terms were based on top GAM model for weight in Table S2.

**Table S5 |** Survivorship from pup emergence to adulthood (1 year) in meerkat (*Suricata suricatta*) offspring. The offspring were from one of three maternal treatment groups: Dominant Control (DC) had naturally greater androgen exposure in utero; Subordinate Control (SC) had naturally lower androgen exposure; Dominant Treated (DT) had experimentally blocked androgen receptors during late gestation.

| Terms | ß | SE | z | *P* |
| --- | --- | --- | --- | --- |
| (Intercept) | 0.50 | 0.82 | 0.06 | 0.952 |
| Maternal treatment: Dominant control | 0.30 | 0.66 | 0.45 | 0.653 |
| *Maternal* *treatment: Dominant treated* | *1.60* | *0.96* | *1.67* | *0.095* |
| **Sex: Male** | **1.20** | **0.59** | **2.01** | **0.044** |
| **Monthly rainfall at emergence** | **-0.03** | **0.01** | **-3.01** | **0.003** |
| Clan size at emergence | 0.02 | 0.04 | 0.56 | 0.575 |

Note. These results derive from a binomial generalized linear mixed model (GLMM) on *n* = 92 individuals. β = the conditional model-averaged estimates. All continuous factors were standardised. Estimates are in reference to: Sex = female; Maternal treatment = subordinate control. Bolding indicates significant terms (*P* < 0.05); italics indicate trends (*P* < 0.10).


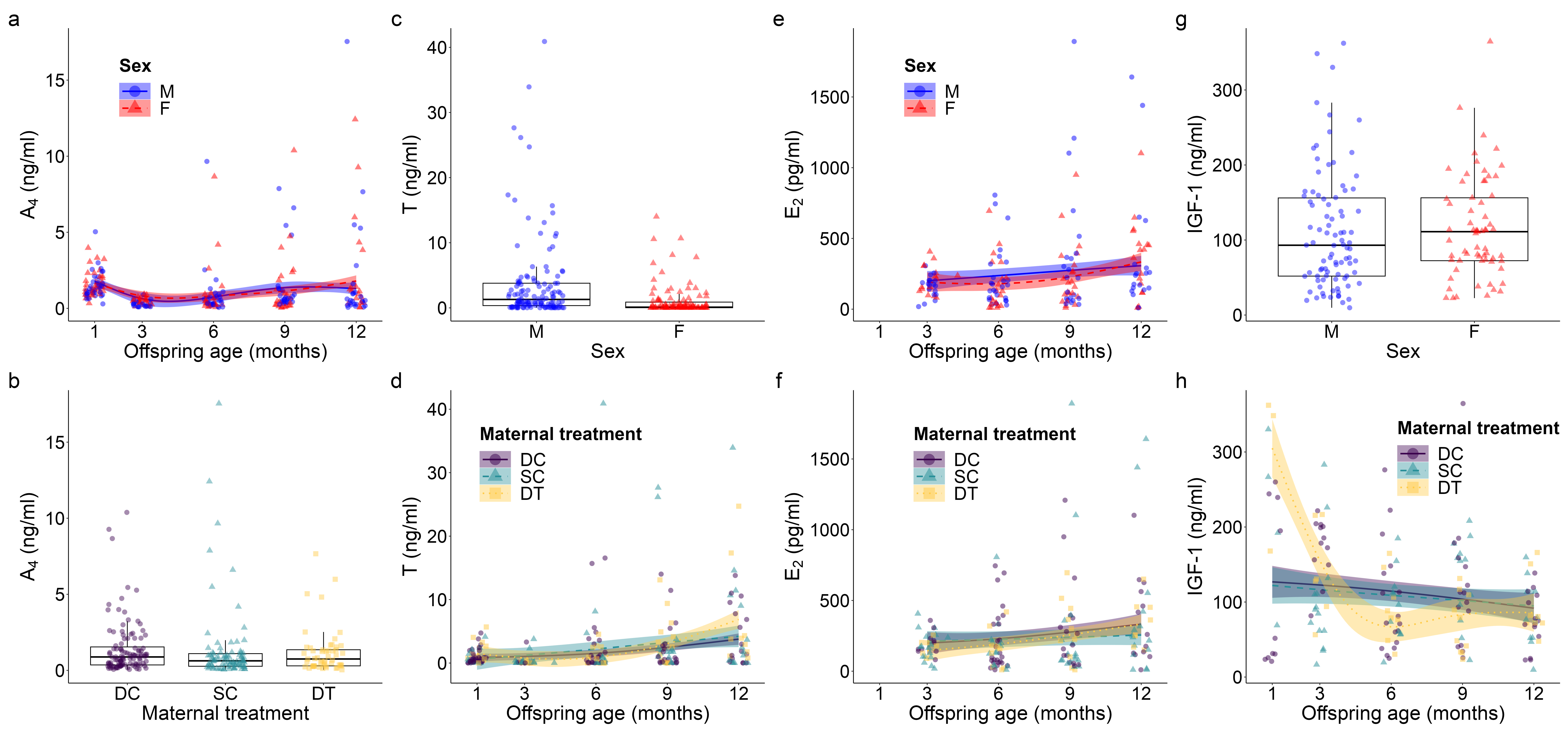


**Figure S1.** Top model-predicted serum concentrations of (a, b) androstenedione or A_4_, (c, d) testosterone or T, (e, f) estradiol or E_2_, and (g, h) insulin-like growth factor 1 or IGF-1 for meerkats by sex (male, M; female, F), age, and/or maternal treatment (dominant control, DC; subordinate control, SC; dominant treated, DT). Sections reprise Figure 1 in the main text, but additionally provide all individual values.


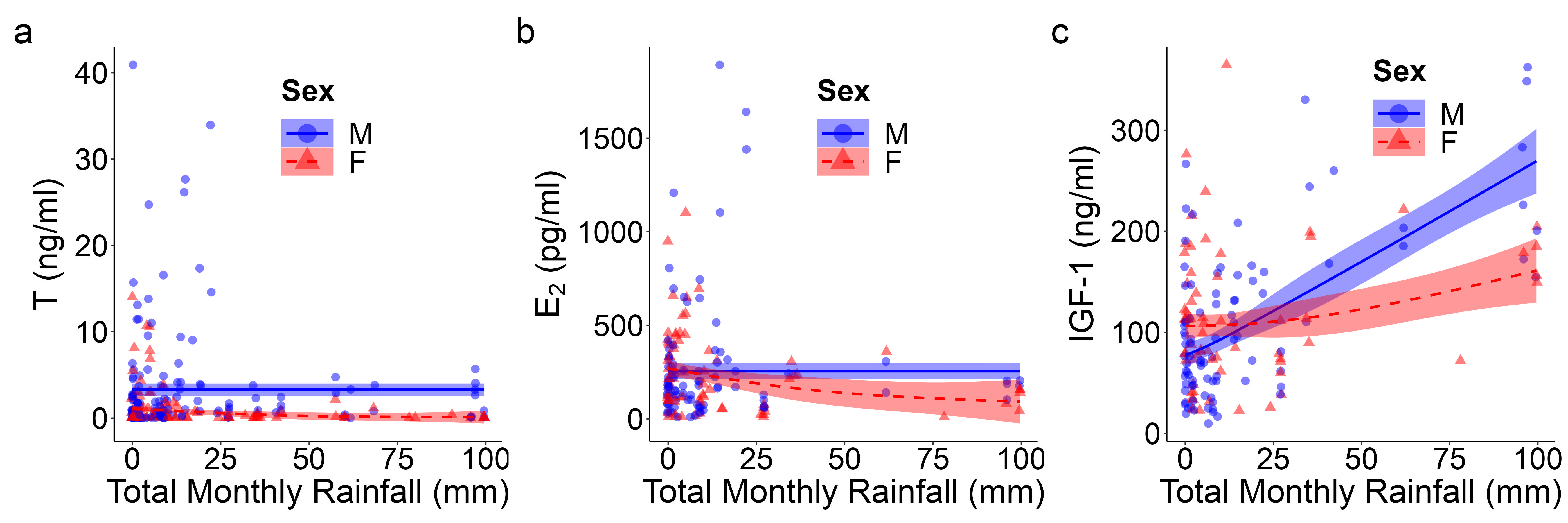


**Figure S2.** Top model-predicted serum concentrations of (a) testosterone or T, (b) estradiol or E_2_, and (c) insulin-like growth factor 1 or IGF-1 for meerkats by sex (male, M; female, F) and total monthly rainfall (mm). Sections reprise Figure 2 in the main text, but additionally provide all individual values.


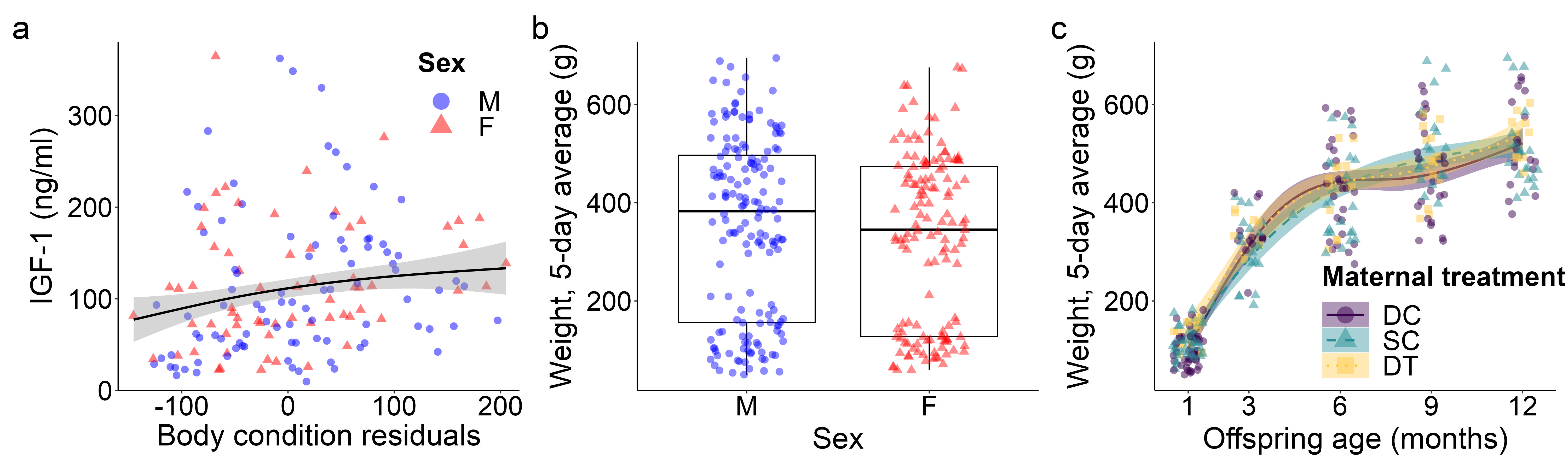


**Figure S3.** Top model-predicted (a) IGF-1 concentrations and (b, c) weights for meerkat offspring. Sections reprise Figure 3 in the main text, but additionally provide all individual values.


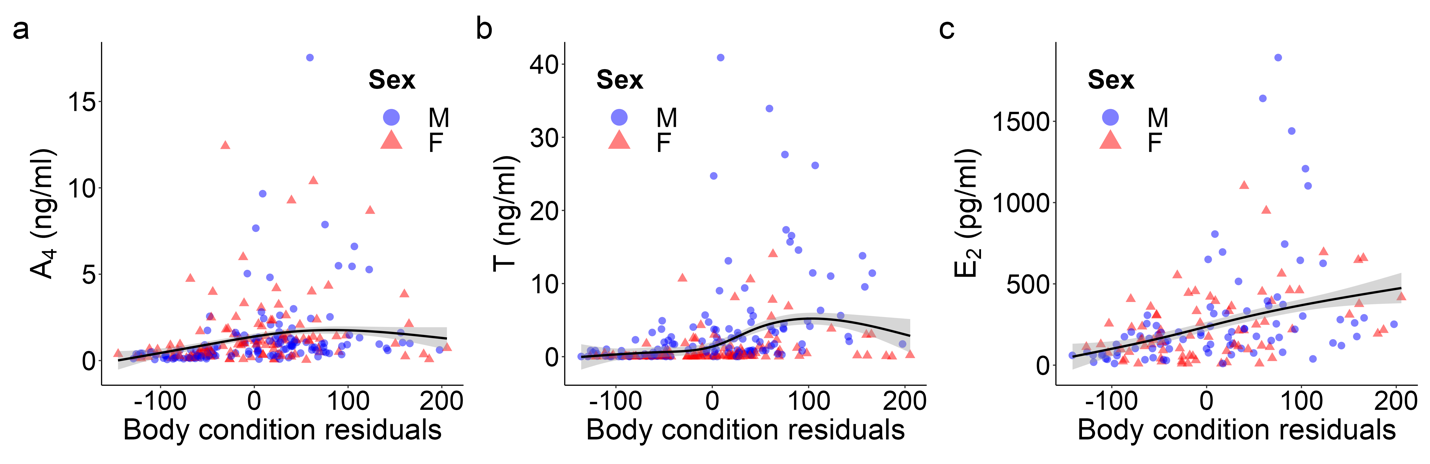


**Figure S4.** Plots of serum hormone concentrations by body condition residuals from top models generated for young meerkats (*Suricata suricatta*), showing (a) androstenedione or A_4_, (b) testosterone or T, and (c) estradiol or E_2_. Raw data are plotted by sex (male, M; female, F) from 1-12 months of age, with a smooth of model estimates and their 95% confidence interval. In (a), a single value of A_4_ (~30 ng/ml) at 1 month of age was excluded for a young male from a dominant control dam (with the highest testosterone concentrations), for ease of visualization.
